# Alternative Splicing in the Differentiation of Human Embryonic Stem Cells into Cardiac Precursors

**DOI:** 10.1371/journal.pcbi.1000553

**Published:** 2009-11-06

**Authors:** Nathan Salomonis, Brandon Nelson, Karen Vranizan, Alexander R. Pico, Kristina Hanspers, Allan Kuchinsky, Linda Ta, Mark Mercola, Bruce R. Conklin

**Affiliations:** 1 Gladstone Institute of Cardiovascular Disease, San Francisco, California, United States of America; 2 Pharmaceutical Sciences and Pharmacogenomics Graduate Program, University of California, San Francisco, San Francisco, California, United States of America; 3 Burnham Institute for Medical Research, La Jolla, California, United States of America; 4 Functional Genomics Laboratory, University of California, Berkeley, Berkeley, California, United States of America; 5 Agilent Technologies, Santa Clara, California, United States of America; University of California Santa Cruz, United States of America

## Abstract

The role of alternative splicing in self-renewal, pluripotency and tissue lineage specification of human embryonic stem cells (hESCs) is largely unknown. To better define these regulatory cues, we modified the H9 hESC line to allow selection of pluripotent hESCs by neomycin resistance and cardiac progenitors by puromycin resistance. Exon-level microarray expression data from undifferentiated hESCs and cardiac and neural precursors were used to identify splice isoforms with cardiac-restricted or common cardiac/neural differentiation expression patterns. Splice events for these groups corresponded to the pathways of cytoskeletal remodeling, RNA splicing, muscle specification, and cell cycle checkpoint control as well as genes with serine/threonine kinase and helicase activity. Using a new program named AltAnalyze (http://www.AltAnalyze.org), we identified novel changes in protein domain and microRNA binding site architecture that were predicted to affect protein function and expression. These included an enrichment of splice isoforms that oppose cell-cycle arrest in hESCs and that promote calcium signaling and cardiac development in cardiac precursors. By combining genome-wide predictions of alternative splicing with new functional annotations, our data suggest potential mechanisms that may influence lineage commitment and hESC maintenance at the level of specific splice isoforms and microRNA regulation.

## Author Summary

The reprogramming of pluripotent stem cells from adult cells is a crucial step toward producing patient-specific cells for transplant therapy. Critical to this goal is the ability to reproducibly drive the differentiation of these cells to specific fates, such as cardiac and neural cells. While gene expression is important in tissue specific differentiation, the impact of alternative splicing on the biology of differentiating cells has not been fully realized. To identify specific splicing events that may determine cell-type-specific differentiation, we compared splicing profiles of human embryonic stem cells (ESCs) and derived cardiac and neural precursors using Affymetrix exon tiling arrays. Segregation of splicing profiles into cardiac-restricted and common cardiac/neural differentiation pattern groups revealed unique groups of genes with clear implications for the biology of cardiomyocyte function and the maintenance of pluripotent ESCs. Alternative splicing of many of these genes, notably regulators of cell death and proliferation, were often predicted to impact protein domain or microRNA binding site inclusion, suggesting that the function or expression of these proteins is altered during differentiation. These results provide further evidence that alternative splicing is important in shaping the functional repertoire of ESCs and differentiated cells.

## Introduction

The differentiation of embryonic stem cells (ESCs) *in vitro* is a powerful system for identifying developmental cues required for lineage commitment. Like their *in vivo* counterparts, the cells of the inner cell mass of the blastocyst, ESCs can self-renew and differentiate into all three adult germ layers. Maintenance of pluripotency and self-renewal depends on the expression of core transcription factors, including Oct4, Sox2, and Nanog. Whole-genome expression [1], microRNA (miRNA) [2], and epigenetic analyses [3],[4] of ESC differentiation have identified additional factors that interact with these core transcription factors to regulate pluripotency. However, the mechanisms that regulate ESC maintenance upstream and downstream of these core regulatory components and the steps required for proper cell fate commitment are poorly understood, largely because of the difficulty of obtaining pure populations of fully differentiated cells and the lack of detailed transcript expression profiles that allow the analysis of transcription and alternative splicing (AS).

Up to 80% of all human genes undergo AS to produce multiple mRNA transcripts that differ in their inclusion of exons and introns [5]. AS often results in unique proteins with biologically distinct compositions and functions [6]. AS can alter domain composition and cellular localization, which can confer distinct signaling properties on the resulting protein. In untranslated mRNA regions, AS can affect RNA stability and localization [6]. Disruption of AS of a single gene can have profound effects on cellular development, ranging from improper neonatal cardiac adaptation [7] to sex-determination [8] and synaptogenesis [9].

Since ESCs can differentiate into all cell lineages, characterizing isoform expression along specific lineage paths requires efficient methods to obtain pure cell populations. To this end, hESCs have been differentiated into neural progenitors (NPs), isolated with an effective neural differentiation protocol, and profiled with whole-genome exon-arrays [10]. This technology can measure the expression of distinct RNA regions and thus identify more complex modes of gene regulation. This analysis revealed AS of serine/threonine kinases and helicases, suggesting that coordinated programs in hESCs direct both cell-type-specific and general differentiation programs. Comparative genome sequence analysis within the vicinity of these AS events revealed putative cis-regulatory sequences that may regulate AS in the differentiation to NPs [10].

While these methods were an important step toward delineating the role of AS in differentiation, profiling of other progenitor cell types and comparisons between cell types is required to identify and understand common processes in differentiation and processes that are specific to different paths of differentiation. Determining the consequences of AS on a genome-wide basis will require tools to predict the effects of AS on protein sequence, domain inclusion, and protein expression.

In this study, we sought to identify AS during differentiation into different progenitor populations by exon-level genome profiling of homogenous populations of undifferentiated hESCs and derived cardiac progenitors (CPs) using a new selectable marker strategy. By comparing CP differentiation to a reported dataset of neural differentiation [10], we identified AS events found only in the differentiation to CPs or the differentiation to both CPs and NPs (common). AS events with common CP-NP or CP-specific patterns produced profound changes in predicted protein domain/motif composition that could affect protein function and expression. Many AS events modified the predicted miRNA binding site composition of transcripts, suggesting that AS may indirectly modulate protein expression.

## Materials and Methods

### Isolation of hESCs and CPs

The genetically engineered hESC lines and electrophysiology of derived CPs have been described in detail in [11]. In brief, H9 ESCs were transduced with a lentiviral preparation that encoded a neomycin-resistance gene controlled by the REX-1 promoter and a puromycin-resistance gene controlled by the α myosin heavy chain promoter. Stable clonal lines were created by neomycin selection of a homogenous population of undifferentiated hESCs. Embryoid bodies were formed in suspension culture for 6 days from hESCs and transferred to gelatin-coated plates. On day 13 of differentiation, embryoid bodies were treated with puromycin for 36 h to isolate CPs. Total RNA from biological triplicates of neomycin-selected undifferentiated hESCs and puromycyin-selected day 40 CPs were extracted with Trizol and prepared for hybridization to human 1.0 ST GeneChip arrays as described [10]. As starting material, ∼1 µg of total RNA was purified with the RiboMinus human transcriptome isolation kit (Invitrogen), cDNA was fragmented and labeled with the GeneChip WT cDNA synthesis and WT terminal labeling kits (Affymetrix), hybridized to individual GeneChip arrays (biological triplicates), and scanned according to the manufacturer's instructions. The data were deposited in NCBI's Gene Expression Omnibus [12] database (GSE13297).

### Additional Microarray Data

Human exon array CEL files for the Cythera NP differentiation datasets (Cy-ESCs and Cy-NPs), HUES6 ESCs, HUES6 NPs, and fetal human central nervous system stem cells were provided by the Gage laboratory (http://www.snl.salk.edu/~geneyeo/stuff/ papers/supplementary/ES-NP) [10]. Exon array data for 11 adult human tissues (testes, spleen, heart, thyroid, muscle, breast, prostate, liver, kidney, pancreas, cerebellum), were downloaded from the Affymetrix website [13].

### Gene Expression Analysis

The methods and program components of AltAnalyze are described in detail in Text S1. For this analysis, AltAnalyze databases (stored as tab-delimited text files) were constructed with build 49 of Ensembl [14] and human genome build 18 of the UCSC Genome Database [15] and Affymetrix annotation files. For all probe sets, expression values and detection above background (DABG) p values were calculated from AltAnalyze using the Robust Multichip Average algorithm [16] by interfacing with Affymetrix Power Tools (APT) [17]. Exon arrays include probe sets that overlap with both exons that are common to nearly all mRNA transcripts for a gene and to those that overlap with only a few mRNA transcripts. Since these common or constitutive exons most likely indicate transcriptional activity of the gene as opposed to a rare isoform, probe sets that overlap with the largest number of distinct mRNA transcripts are considered constitutive and thus most informative for calculating gene expression values. The number of mRNAs that overlap with a probe set was obtained from the Affymetrix annotations file HuEx-1_0-st-v2.na23.hg18.probe set.csv. Only probe sets that overlap with a single Ensembl gene (based on the start and end genomic coordinates of the probe set and gene) are considered in AltAnalyze. For each Ensembl gene and for each microarray, gene expression is determined by calculating the mean expression of all constitutive probe sets. If no constitutive probe sets are present for an Ensembl gene, gene expression is calculated by using the mean of all exon-associated gene-linked probe set intensities. Complete gene expression results are provided in Dataset S1.

### Alternative Exon Analysis with AltAnalyze

**Figure 1:**
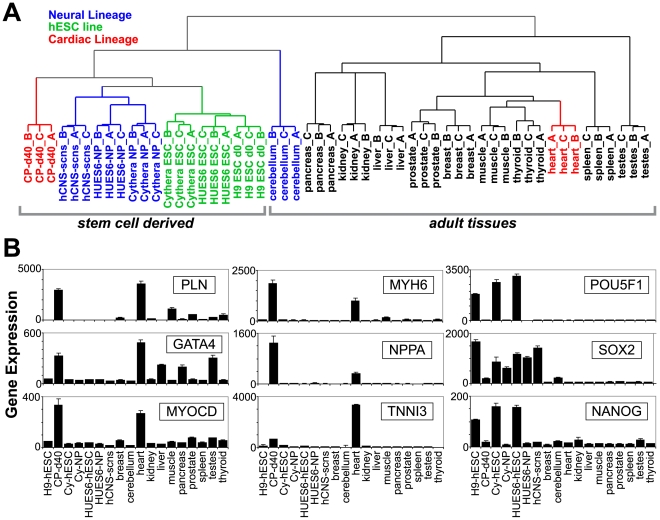
Comparison of hESC differentiation and adult tissue array profiles. Human Affymetrix exon array data were compared for REX+ hESCs and derived CPs; Cythera, HUES6 hESCs and derived NPs; fetal human central nervous system stem cells (hCNS-scns); and 11 adult tissues, processed by RMA together. (A) Relative changes in gene expression (log2 fold, relative to the global expression mean) for all samples were clustered by array (rather than genes) for any Ensembl gene with a relative change in gene expression >2. Biological triplicates are indicated for each tissue or cell line. (B) Gene expression profiles for this combined dataset and for specific markers of CP-specification (columns 1 and 2) and for pluripotency (column 3).

To identify alternative exons, AltAnalyze was run with default parameters. This analysis consists of (A) selecting microarrays for expression summarization with RMA, (B) defining biological groups for each array and pairs of groups for alternative exon analysis (e.g., hESCs and CPs), (C) downloading/loading appropriate library and annotation files for the microarray, (D) defining thresholds for probe set filtering, (E) defining thresholds for alternative exon analysis statistics (splicing-index and MiDAS [18]), (F) determining methods used to identify predicted domains/motifs and miRNA binding sites predicted to be regulated, (G) running the analysis, and (H) exporting result files (Text S1). We performed two different sets of array normalizations. The first included all aforementioned cell lines and tissues, and the second included only cell lines. The combined cell line and tissue normalization was only used for combined gene expression comparison analyses (Figure 1) and comparison of tissue and cell line splicing patterns. The cell-line-specific normalization was used for all remaining downstream CP and NP alternative exon analyses.

After RMA expression values and gene expression statistics (e.g., hESC and CP group gene expression averages, fold changes, and *t* test p values) are exported, AltAnalyze filters probe sets to identify those that align to a single Ensembl gene and that match user-defined expression and DABG p value thresholds (Text S1). Only probe sets with a DABG p<0.05 and a non-log expression value >70 [19] are retained for further analysis of all hESC–progenitor pair-wise comparisons. Using the splicing index (SI) method [19],[20], AltAnalyze calculates the likelihood and extent of AS for all Ensembl genes with one or more constitutive probe sets. Two probability estimates for alternative exon regulation are calculated with a one-way analysis of variance model, MiDAS [19], by interfacing with APT (version 1.4.0) and SI, by performing a *t* test of the normalized exon expression values (exon probe set expression divided by constitutive expression) for the control and experimental sample groups.

The primary filters for identifying alternative exons were a conservative SI fold change >1 (equivalent to a 2-fold difference in expression relative to constitutive expression levels), an SI *t* test p value <0.05, a MiDAS p<0.05, and constitutive gene-expression fold change <3 (absolute). Genes with a constitutive fold change >3 were excluded to remove potential false-positive alternative exons that arise when only constitutive probe set variance is observed. For this analysis, we considered only AltAnalyze ”core” probe sets and probe sets overlapping with any mRNA contained within the AltAnalyze mRNA database (Ensembl or UCSC Genome Database).

To visualize alternative exons in the context of genes, we wrote a prototype plugin, currently in development, for the network visualization software Cytoscape [21]. This plugin, SubgeneViewer, allows colors indicating inclusion or exclusion of exons to be mapped onto exon and splicing structures that can be selectively viewed from protein interaction networks or pathways.

### Identifying Alternative Protein Domains and Motifs with AltAnalyze

**Figure 2:**
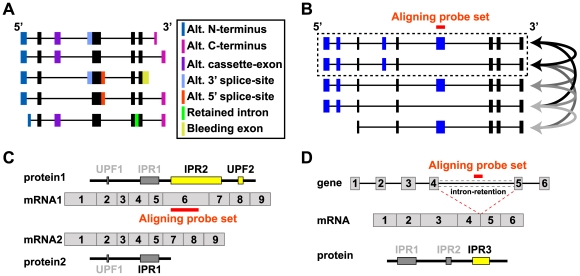
Assigning AltAnalyze mRNA and protein annotations. Theoretical transcripts with distinct exon compositions are shown. (A) Distinct alternative (Alt.) exon annotations for five mRNA transcripts, where the filled boxes are sequences retained in the processed mRNA transcript. Black filled boxes are exons common to all isoforms (constitutive). AltAnalyze considers all alternative exon annotations as AS except for alternative-N-terminal exons (expressed through alternative promoter selection). (B) All pairs of mRNA transcripts that do or do not align to an exon array probe set are compared to identify a single pair of competitive isoforms that minimally differ in exon composition. Curved arrows indicate all possible competitive transcript comparisons. The top selected competitive isoforms (dashed box) have the fewest exon differences and have the most exons in common. AltAnalyze selects this transcript pair for analysis of downstream protein domain/motif composition, after corresponding protein sequences are selected. (C) Protein domains and motifs differing between competitive isoforms. Exons for the two transcripts are labeled in order, 5′ to 3′, with protein sequence and Uniprot features (UPF) or InterPro regions (IPR) corresponding to each exon displayed above or below them. Yellow filled boxes indicate domains and motifs differencing between the competitive isoforms. (D) Domains and motifs directly aligning to a probe set's genomic position. A theoretical probe set aligning to the intron of a gene is shown. InterPro domains/motifs whose genomic position (genomic exon start and exon end position) overlaps with a given probe set (genomic start and end position) are shown with a yellow filled box. Rather than comparison of two protein sequences with the competitive isoform analysis, only a single protein sequence is required for the direct genomic alignment method.

To identify protein domains and motifs potentially modified/disrupted by AS, a series of databases is built with each build of AltAnalyze (stored as distributed text files). These databases consist of an aligning and nonaligning protein (competitive isoforms) for all probe sets (Figure 2B), corresponding protein sequences, relative comparison information for the two competitive alternative protein isoforms (e.g., alternative-N-terminal, alternative-C-terminal sequence) (Figure 2A), protein domains/motif sequences found in one protein but not the other (Figure 2C), and domains/motifs whose genomic position overlaps with any probe set (Figure 2D). In short, competitive protein isoforms are selected by identifying exons from Ensembl or the UCSC Genome Database that overlap with a probe set based on genomic position, identifying mRNA competitive transcripts that contain or do not contain that exon, comparing the exon structure of all possible competitive transcripts and identifying a single competitive transcript pair that contains the fewest combined distinct exons (exons unique to either transcript) and the most exons in common to both. Corresponding proteins for selected competitive isoforms are identified from Ensembl and NCBI's web services [22] or are derived by *in silico* translation. Protein domain and motif sequences are obtained directly from UniProt's [23] sequence annotation features or from InterPro [24] sequence annotations (alignment e-value <1) for every Ensembl protein. AltAnalyze reports any InterPro sequences with a description field or any UniProt sequence annotation feature that is not of the type CHAIN, CONFLICT, VARIANT, VARSPLIC, or VAR_SEQ. The competitive protein isoform analysis only considers the alternative exon of interest and does not attempt to combine information from other potential alternative exons. Only InterPro genomic start and end positions are extracted to determine genomic coordinate overlap with probe sets.

In addition to these protein annotations, putative miRNA binding sites from PicTar [25], miRanda (http://www.microrna.org), miRbase [26] and TargetScan (http://www.targetscan.org) within probe set consensus sequences are stored in a database for use by AltAnalyze. Protein and miRNA binding site annotations for all alternatively regulated probe sets (alternative exons) are reported in the AltAnalyze results files and are assessed for over-representation. Over-representation is assessed by calculating an over-representation z score, a permutation based p value (derived by re-running the z score analysis 2000 times with random probe set inputs) and a Benjamini-Hochberg [27] adjusted p value of the permutation p to take account for multiple hypothesis correction (Text S1). For all domain/motif and miRNA over-representation analyses, only elements (domain, motif, or miRNA binding site) with a z score >2, Benjamini-Hochberg p<0.05 and three or more genes associated with the element are reported. To test for differences between the number of miRNA binding sites up- and downregulated in hESCs versus CPs, the number of genes with alternative exons up- and downregulated and the number of genes with these patterns of expression and predicted miRNA binding sites are evaluated with a χ^2^ test.

### Analysis of Variance

To segregate transcriptionally regulated genes and AS events into CP-specific and common CP-NP differentiation patterns, we used a two-way ANOVA strategy in which the LIMMA package in Bioconductor [28] was used to compare day 40 CP arrays with NP arrays along with their respective pluripotent hESC controls. The Cythera hESC line dataset was used to examine NP differentiation, which had smaller sample-to-sample variability than the HUES6 hESC line dataset when analyzed with RMA (data not shown). Each differentiated condition (CP and NP) was normalized to the mean of its hESC reference set and used to calculate a p value to assess whether NP and CP profiles had a common CP-NP or CP-specific differentiation pattern (termed here as a differentiation or interaction effect, respectively). The differentiation p value indicates the likelihood that CP and NP differentiation have a common expression pattern, whereas the interaction p value indicates the likelihood that CP and NP differentiation have a dissimilar expression pattern for a given exon. For both patterns, a fold difference (log2) >1 was required for CP vs. REX+ hESCs. Additionally, for the common CP-NP differentiation pattern group, a fold difference (log2) >0.5 was required for Cythera NP vs. Cythera hESCs (in the same direction as the CP comparison); and for the CP-specific group, a fold difference (log2) <0.5 (in the same direction as the CP comparison) was required.

### Pathway Analysis

Gene Ontology (GO) and pathway over-representation were evaluated with the program GO-Elite (http://www.genmapp.org/go_elite) [29]. Unlike traditional GO analysis programs, GO-Elite prunes out related ontology terms to report a single significant term along each branch of the hierarchy when multiple significant terms are present. GO-Elite also analyzes pathways from http://WikiPathways.org [30]. GO-Elite uses an over-representation analysis similar to that applied by AltAnalyze to domains and other elements. Only GO terms and pathways with a z score >2, a permutation p<0.01, and three or more regulated genes for the pathway/GO-term were reported. Complete pathway analysis results are provided in Dataset S4.

### Confirmation of Alternative Exon Expression

Alternative exons were selected for confirmation with RT-PCR using the following criteria: (1) prior evidence of AS or presence of predicted miRNA binding sites, (2) a small number of alternative exons per gene, and (3) predicted domain/motif changes. The second criterion was applied to favor splice events where both isoforms could be amplified in a single reaction and where domain/motif-level changes could be attributed to the splicing event examined. Fifty alternative exon sequences were selected for confirmation. Optimal flanking, isoform-specific, or constitutive primers designed with a custom implementation of primer 3 called AltPrimer (http://conklinwolf.ucsf.edu/tools/ picoprimer.html). For RT-PCR, total RNA was diluted to ∼10 ng/µl and amplified with the OneStep Superscript III RT-PCR kit (Invitrogen) for 28, 35, or 40 cycles at annealing temperatures of 55 or 58°C using isoform-specific or constitutive flanking primers. The reaction products were separated on a 2–2.5% DNA-agarose gel and stained with ethidium bromide. An RT-PCR reaction that produced the correct amplicon sizes was considered to confirm alternative exon expression. Primer sequences are available in a Table S1.

## Results and Discussion

### Comparison of hESC-Derived Cardiac and Neural Progenitors to Adult Tissues

To identify genes with common CP-NP differentiation or CP-specific AS patterns, we isolated homogenous populations of hESCs and cardiac precursors and compared them to a dataset of neural precursor differentiation [10]. To determine the relationship between undifferentiated, progenitor and adult tissues, we performed an unbiased analysis using expression clustering and an analysis of cell-specific marker gene expression.

Homogenous populations of undifferentiated hESCs and CPs were isolated by modifying the H9 ESC line to stably express neomycin-and puromycin-resistance genes, driven by the pluripotent-cell-specific REX-1 and CP-specific myosin heavy chain alpha (MHCα or MHY6) promoters, respectively (see Materials and Methods). In embryoid bodies that underwent puromycin selection and were differentiated for 40 days, the action potentials and axial force measurements of the resulting CPs were similar to those of normal fetal cardiomyocytes [11]. RNA harvested from REX+ hESCs and CPs was analyzed with Affymetrix human exon 1.0 arrays; these data were combined with a dataset of two published hESC lines (Cythera and HUES6) and NPs derived from these hESCs [10] and a dataset of 11 adult human tissues [13].

Gene expression values from these exon arrays were determined for constitutive aligning or all probe sets for each Ensembl gene (Materials and Methods) (Dataset S1). As shown by hierarchical clustering [31] of these gene expression profiles, the three hESC lines (H9, Cythera, and HUES6) were more closely correlated to each other than to their differentiated progenitors or adult tissues (Figure 1A). However, CPs and NPs, while more closely correlated to each other and undifferentiated hESCs, were less correlated to their *in vivo* tissue counterparts (heart and cerebellum). For several reasons, such differences are not unexpected; adult tissues are composed of a variety of cell types, passaged cell lines were compared to adult cells, the arrays were processed in different laboratories, and the cells are of distinct genetic origins. Although gene expression profiles of CPs were not closely correlated with samples from adult heart, CP gene expression levels were similar to those of adult heart for all cardiac markers examined (Figure 1B). Both NP lines express neuron-specific markers [10]. Thus, these progenitor cells are closely related to hESCs but retain cell-type-specific marker expression and therefore are appropriate cell systems for assessing AS and differentiation into cardiac and neural lineages.

### AS Is a Key Feature of CP Differentiation

To identify alternative exons in day 40 CPs versus REX+ hESCs and link the results to predicted sequence changes that might alter protein expression/function, we created a free, open-source application called AltAnalyze (http://www.AltAnalyze.org) (Materials and Methods). For exon array analysis, AltAnalyze uses the SI approach [19],[20] to calculate a probe set fold change corrected for gene-expression and corresponding SI *t* test and MiDAS p values. To identify higher-confidence alternative exons, only regulated probe sets overlapping with exons in annotated mRNAs (Ensembl or UCSC Genome Browser, including retained introns) were used for further analyses.

Of the 13,583 genes with evidence of expression, 16% (2,106) were predicted to have at least one alternative exon in the differentiation to CPs, as compared to 3,044 genes with up- or downregulated gene expression (Table 1). Of the alternatively regulated genes (ARGs) containing one or more alternative exons, 42% (876) had alternative exons with prior evidence of AS (defined here as alternative or mutually exclusive cassette exons, alternative splice sites, exon-bleeding [15], alternative-C-terminal exons or retained introns; see Figure 2A), 8% (170) had an alternative promoter (AP), and 7% (152) had both; the remainder occurred in constitutive exons (Dataset S2). Thus, our analysis predicts that 3% of all Ensembl genes examined (876 of 29,151) are alternatively spliced relative to 10% (3,044 of 29,151) differentially expressed during the differentiation of hESCs to CPs.

**Table 1:** Alternative gene regulation during CP differentiation.

	*Genes (n)*	*Genes examined (n)*
**Differentially expressed genes**	**3,044**	29,151
**Genes with alternative exons (ARGs)**	**2,106**	13,583
Alternative splicing (AS)	876	
Alternative promoter (AP)	170	
AS and AP	152	
No evidence of AS or AP	908	

Number of genes differentially expressed and alternatively spliced. Gene expression values were calculated for 29,151 Ensembl gene identifiers, of which only 13,583 were examined for AS. Genes examined for AS were required to have constitutive annotated probe sets expressed in both undifferentiated H9 ESCs and derived CPs. Transcriptional activity of genes was determined by using either constitutive probe sets, if present, or all probe sets, when not present. Genes with alternative exons are unique Ensembl genes reported by AltAnalyze with alternatively expressed probe sets. Genes with alternative exons only aligning to AS annotations or only to AP annotations are reported along with genes that associate with both AS and AP (multiple exons or one exon with multiple annotations) and no evidence of AS or AP.

As in earlier studies of NP differentiation [10], pathway analysis of all alternative exons showed that serine/threonine protein kinases and genes with helicase activity were highly enriched during differentiation to CPs. We also found over-representation of actin cytoskeleton, RNA splicing, cell-cell adherens junction, chromatin binding, regulation of muscle contraction, and cell cycle checkpoint genes, among others, using the program GO-Elite (Dataset S4).

### Assessing the Impact of AS on Protein Domain and miRNA Binding Site Architecture

Although analysis of protein composition determined by AS is not entirely novel [32],[33], AltAnalyze has many novel features. For example, it incorporates protein, domain, and motif information from multiple protein annotation databases (UniProt, InterPro), detailed information on the competitive isoforms analyzed, and analyses of miRNA binding site predictions from multiple databases (PicTar, miRbase, miRanda and TargetScan). By default, domain/motif predictions are derived by comparing two protein isoform sequences—one that aligns to the alternative exon and another in which the exon is absent from the corresponding mRNA sequences (competitive isoforms). Since many aligning and nonaligning mRNAs can exist for each alternative exon, an algorithm is used to identify isoform pairs with the smallest differences in exon composition (Figure 2B). Identification of the corresponding protein (Ensembl or NCBI) or *in silico* translation of these competitive isoforms allows AltAnalyze to identify protein domain or motif sequences (InterPro and UniProt) that differ in the inclusion of a target exon between the two isoforms (competitive isoform analysis) (Figure 2C). An alternative method analyzing domain/motif regulation is also used, whereby the genomic coordinates of probe sets that correspond to an alternative exon are compared to the genomic position of all InterPro domains and motifs for that gene (direct genomic alignment) (Figure 2D and Text S1). Since AltAnalyze is a freely distributed software package, these analyses can easily be applied to any exon-array dataset.

### AS Modifies Predicted Protein Domain and miRNA Binding Site Architecture during Cardiac Differentiation

For alternative exons regulated during CP differentiation, predicted changes in domain/motif and miRNA binding site composition were examined with AltAnalyze. The majority of alternative exons during CP differentiation (79%), corresponded to competitive mRNA isoforms (sharing some exons, but not the probed exon) (Materials and Methods) (Dataset S2). Competitive isoform analysis predicted that one or more protein domains/motifs would be modified or absent in 62% of alternative exons. But with the direct genomic alignment method, only 32% of the alternative exons were predicted to affect domains/motifs. Although 27% of the direct genomic alignment predictions only occurred with this prediction method, these typically occurred in constitutive regions with no competitive isoforms identified. Thus, many of the domain/motif changes predict by competitive isoform analysis occurred as result of indirect protein differences (e.g., protein truncation), while others could only be identified with the direct genomic alignment method.

To determine whether certain domains or motifs were over-represented by both methods, we examined the associated over-representation z scores and permutation-based p values for all CP differentiation ARGs (Materials and Methods). Using either the competitive isoform or direct genomic alignment methods, AltAnalyze identified over-representation of the laminin globular domain, myosin head motor region, and spectrin, plectin, collagen triple helix and rho-binding repeats. In addition, competitive isoform analysis alone identified the KCNQ, C-terminal and metalloprotease regions, and the actin-binding, spectrin-actin binding, KH, CUB, FERM, IQ, SH3, and protein kinase domains. Direct genomic alignment identified the START lipid-binding, semaphorin and Dbl homology domains, among others. Several of these same elements were also enriched with the AltAnalyze analysis of NP differentiation, including spectrin repeats and CUB, FERM, IQ, SH3, and the laminin globular and actin-binding domains (Datasets S2 and S3).

In addition to protein domains/motifs, 12.5% of ARGs associated with CP differentiation (264 of 2,106) resulted in the predicted gain or loss of at least one miRNA binding site (Table 2). Nearly half of the miRNA binding site changes (129 of 264) were in an exon with evidence of AS, often as a result of intron retention, exon bleeding, or an alternative C-terminus; the remainder were in a constitutive exon with a variable 3′ length and thus are not likely due to AS (Dataset S2).

**Table 2:** Regulation of miRNA binding sites during CP differentiation.

	*Genes (n)*	*Total genes (n)*
**Differentially expressed miRNAs**	**26**	216
**ARGs with miRNA binding sites**	**264**	11,085
Upregulated in hESCs	202	
Downregulated in hESCs	60	
Up- and downregulated in hESCs	2	

Transcriptionally regulated genes annotated as miRNAs and genes containing alternative exons overlapping with predicted miRNA binding sites. Analysis of gene transcription data from the Affymetrix exon array, highlights 26 Ensembl annotated miRNA genes differentially expressed with CP differentiation (up- or downregulated >2 with a *t* test p<0.05) out of 216 probed on the array and in Ensembl. Of the 13,583 genes analyzed for alternative exon expression, 11,085 had probe sets containing at least one predicted miRNA binding site. The pattern of alternative exon expression is indicated for hESCs relative to CPs. Upregulation indicates that the alternative exon containing a putative miRNA binding site is expressed at a higher level in hESCs than CPs, relative to constitutive expression levels. Genes with up- and downregulation of miRNA binding sites indicates that more than one alternative exon with miRNA binding site(s) was present having opposite expression patterns.

Interestingly, a recent study also observed alternative expression of exon regions containing miRNA binding sites, when these cells began to actively divide [34]. This study was performed with the mouse Affymetrix exon array and revealed shorter untranslated regions (UTRs) and fewer miRNA binding sites in the mRNAs of proliferating CD4 positive T lymphocytes. Similarly, fewer predicted miRNA binding sites were found in pluripotent REX+ hESCs than in CPs (204 downregulated alternative exons with binding sites in hESCs out of 264, χ^2^ p<0.01). The same trend was observed for NPs versus hESCs (Datasets S2 and S3). Thus, a loss of miRNA binding sites may be common to actively dividing cells. Using the same analysis applied to domains and motifs, only one miRNA binding site was over-represented among ARGs in CP differentiation (hsa-miR-219) and only one for NP differentiation (hsa-miR-487a). Neither miRNA has previously been associated with ESC differentiation.

### AltAnalyze Identifies Known Differentiation Splicing Events That Correlate with Altered Protein Function

**Figure 3:**
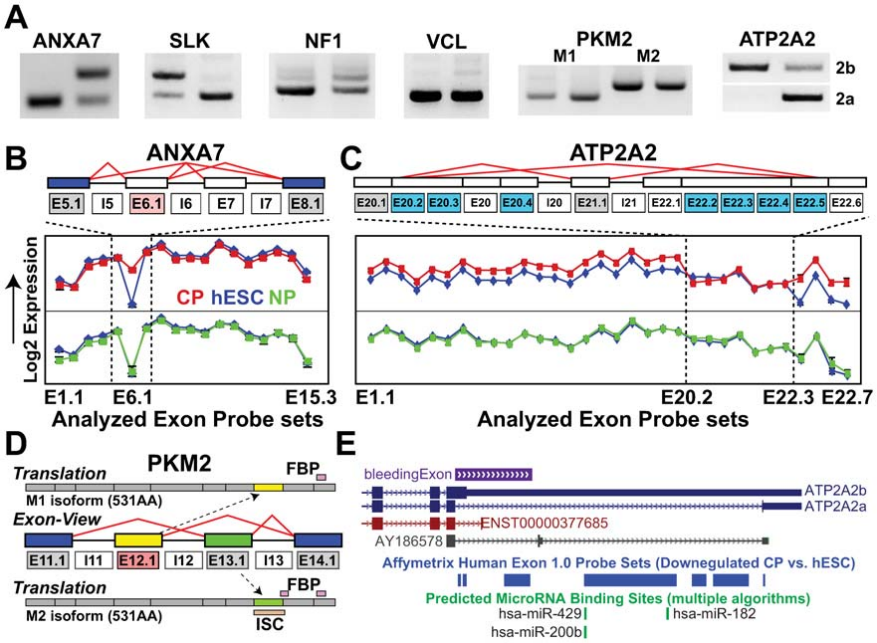
Analysis of verified AS events identifies novel functional associations. (A) Expression of splice isoforms confirmed by RT-PCR of genes with prior evidence of AS. ANXA7, SLK, NF1, and VCL were confirmed with flanking primers, and PKM2 and ATP2A2 with isoform-specific primers. DNA agarose gel images, with REX+ hESCs RNA on the left side of the gel and CPs on the right. (B–C) Exon structure (top graphic) and expression profiles (bottom graphic) for ANXA7 and ATP2A2. (B) SI fold changes are shown for probe sets aligning to exons and introns in the prototype Cytoscape plugin SubgeneViewer. Light red boxes indicate upregulation for CP versus hESC; blue boxes, downregulation; gray boxes no significant change; white boxes no probe set detected above expression thresholds. Probe set expression values (log2) are displayed for both CP (top graphs) and NP differentiation (bottom graph), ranked in order of genomic position on the *x*-axis. Blue data points indicate hESC expression; red data points indicate CP expression; green data points indicate NP expression. (D) Domain/motif annotations for each PKM2 alternative isoform (M1 and M2). The two mutually exclusive isoforms produce proteins differing in the predicted inclusion of an FBP binding region and intersubunit contact (ISC) sequence as defined by UniProt. Yellow and green mutually exclusive exons are shown relative to the translated position of these exons in resulting proteins. (E) miRNA binding sites that overlap with the last intron of ATP2A2. Exons for ATP2A2 transcripts (solid dark blue, red, and black boxes) are displayed 5′ to 3′ (forward strand) along with UCSC splicing annotations (purple box), and aligning probe sets, downregulated in CPs versus hESCs (blue boxes). These downregulated probes sets correspond to those shown in panel C. ATP2A2 isoforms 2a and 2b are indicated. The term ”multiple algorithms” indicates that two or more miRNA binding site prediction algorithms (PicTar, miRanda, miRbase or TargetScan) predicted a binding site in aligning probe sets.

Several predicted splicing events identified in CPs were verified in previous analyses of hESC differentiation. These included genes that underwent AS in the differentiation to NPs (SLK, SORBS1 [10], and NFYA [35]) and in cardiac/muscle differentiation (ATP2A2 [36],[37], NF1 [38], PKM2 [39], and ANXA7 [40]). SLK had one of the largest exon inclusion SI scores in hESCs, and ANXA7 had one of the largest in CPs. RT-PCR analysis of six of these AS events (ANXA7, ATP2A2, NF1, PKM2, SLK, and VCL) in REX+ hESCs and CPs verified the expected changes in isoform expression (Figure 3A–C).

At least three of these verified AS events correspond to modified protein function/expression, producing differences in cell metabolism (PKM2) [41],[42], signaling (VCL) [43], or mRNA stability (ATP2A2) [36]. In each case, AltAnalyze predicted the regulation of protein domains or motifs previously associated with these differences. In the case of PKM2 or pyruvate kinase, two isoforms (M1 and M2) are expressed through mutually exclusive splicing (mutual gain and loss of a cassette exon) [39]. Although the two alternatively spliced exons are the same length (167 base pairs) and have 60% protein sequence identity to each other, they differ in their tissue developmental expression patterns, protein motif composition, and *in vivo* functions [39]. M1 is largely present in normal adult heart, skeletal muscle, and brain and is not allosterically regulated by fructose-1,6-bisphosphate (FBP). M2 is present only during embryonic development and in tumors, is regulated by FBP, and promotes proliferation [41],[42]. Isoform expression levels and protein predictions by AltAnalyze matched the previously identified motif changes corresponding to functionally relevant differences in these proteins (loss of the UniProt-defined FBP binding region and intersubunit contact and upregulation of the M1 exon in CPs) (Figure 3D).

For vinculin (VCL), the gain of a vinculin/alpha-catenin sequence by AS is associated with altered ligand binding properties of the muscle form of the protein [43]. For this same alternative exon, AltAnalyze predicted the same previously reported protein sequence difference. For ATP2A2 (cardiac sarco/endoplasmic reticulum calcium ATPase), intron retention of 4068 base pairs before the last exon increases the length of the cytoplasmic topological domain (45 amino acids [aa]) and 3′ UTR. Expression of the long C-terminal form (isoform 2b) of ATP2A2 (hESC-enriched) increases mRNA degradation of this transcript *in vitro* [36]. AltAnalyze similarly predicted that this AS event would increase the length of the cytoplasmic topological domain (UniProt), in addition to the inclusion of several putative miRNA binding sites (hsa-miRNA-429, 200b, and 182), each supported by evidence from multiple miRNA binding site prediction algorithms (Figure 3E). CPs had decreased expression of the retained intron containing these miRNA binding sites relative to hESCs. Interestingly, two of these miRNAs, miRNA-200b and miRNA-182, were highly enriched in CPs derived from mouse ESCs [44], suggesting a possible mechanism for the increased degradation of the long 3′UTR form. Thus, AltAnalyze suggested a new mechanism for the regulation of ATP2A2 expression by AS.

### Regulation of Distinct Pathways for Cardiac- and Differentiation-Associated Splicing Events

The combination of cardiac and neural differentiation data provides a unique opportunity to define molecular profiles unique to or in common to specific differentiation paradigms. To identify AS events during CP differentiation that correspond to CP-specification or inhibition/promotion of differentiation, we used two-way ANOVA to compare alternative isoform expression between cardiac and neural differentiation (Materials and Methods). Considering the large number of well-characterized cardiac, neural, and hESC markers, we initially applied this method to transcriptionally regulated genes for the two differentiation paradigms. Of 3,044 differentially expressed CP genes, 1,962 had a common CP-NP differentiation expression pattern in cardiac and neural differentiation (differentiation p<0.05), and 951 were preferentially regulated during CP differentiation (interaction p<0.05). As predicted, genes with the lowest ANOVA differentiation (common to CP and NP differentiation) p value corresponded to key pluripotency factors (e.g., LIN28, OCT3/4) and pathways (cell cycle control and regulation of pluripotency). Likewise, genes with the lowest ANOVA interaction (differing in CP and NP differentiation) *p* value corresponded to well-described cardiac markers (e.g., TNNC1, TNNI1, TNNI3, MYH6, MYH7, PLN, GATA4, GATA6, NPPA, TBX5, TBX20) and pathways (early cardiac developmental, muscle proliferation, cardiac muscle contraction) when analyzed with GO-Elite (Figure S1).

**Figure 4:**
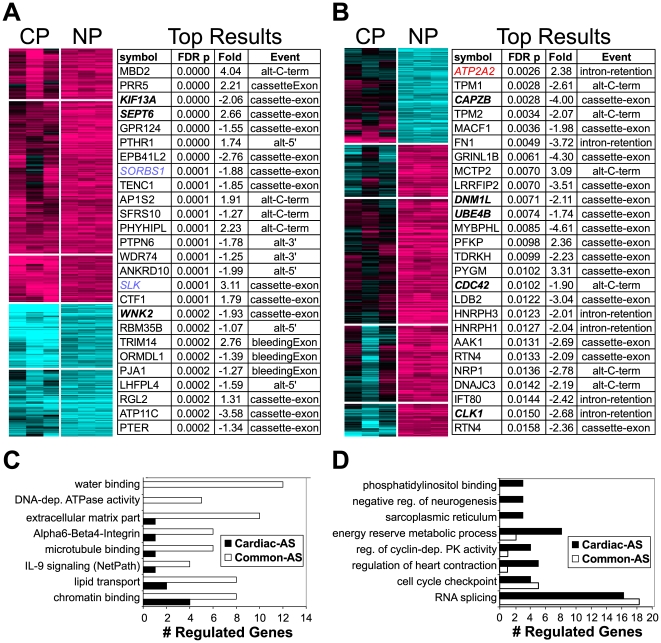
Genes with common CP-NP or CP-specific AS patterns associate with distinct pathways. AS predictions with evidence of (A) a common CP-NP differentiation or (B) a CP-specific expression pattern, relative to undifferentiated hESCs. Adjacent to each heatmap are alternative exons, ranked according to the ANOVA false-discovery rate (FDR) p value. Next to this p value, are the SI fold changes reported by AltAnalyze (negative values indicate increased alternative exon expression in CPs and vice versa). Gene names in blue have prior evidence of AS during hESC differentiation; genes in red have prior evidence of AS during cardiac differentiation. Genes associated with GO terms and WikiPathways are graphed that are overrepresented in genes with a (C) common CP-NP or (D) CP-specific AS pattern.

When applied to alternative exons regulated during CP differentiation, this ANOVA method identified 565 genes with a common CP-NP differentiation expression pattern and 414 genes with a CP-specific expression pattern (Figure 4A, B). In both groups, we considered only alternative exons associated with AS annotations by AltAnalyze. Three AS events identified from previous studies (SORBS1, SLK, and ATP2A2) were among the top 26 ranked genes (ANOVA p) in the two expression pattern groups examined.

When pathway analysis was applied to AS genes with a common CP-NP differentiation splicing pattern, the most enriched ontology categories/pathways were water binding, RNA and chromatin binding, integrin-mediated signaling, microtubule binding, extracellular matrix, and lipid transport (Figure 4C). In contrast, AS genes with a CP-specific pattern were enriched in pathways for phosphatidylinositol binding, sarcoplasmic reticulum, negative regulation of neurogenesis, regulation of heart contraction, Wnt receptor signaling, and regulation of cyclin-dependent protein kinase activity. Both sets were enriched in serine/threonine kinases, helicases, actin cytoskeletal, RNA splicing, and cell cycle checkpoint genes (Figure 4D and Dataset S4). These results imply that the loss of pluripotency corresponds to AS of genes that regulate transcription, cell-cell contact formation, and metabolism, while cardiac-enriched events favor contractile pathways, inhibition of neurogenesis, and Wnt signaling (Dataset S4).

### Confirmation of CP Differentiation AS by RT-PCR

**Figure 5:**
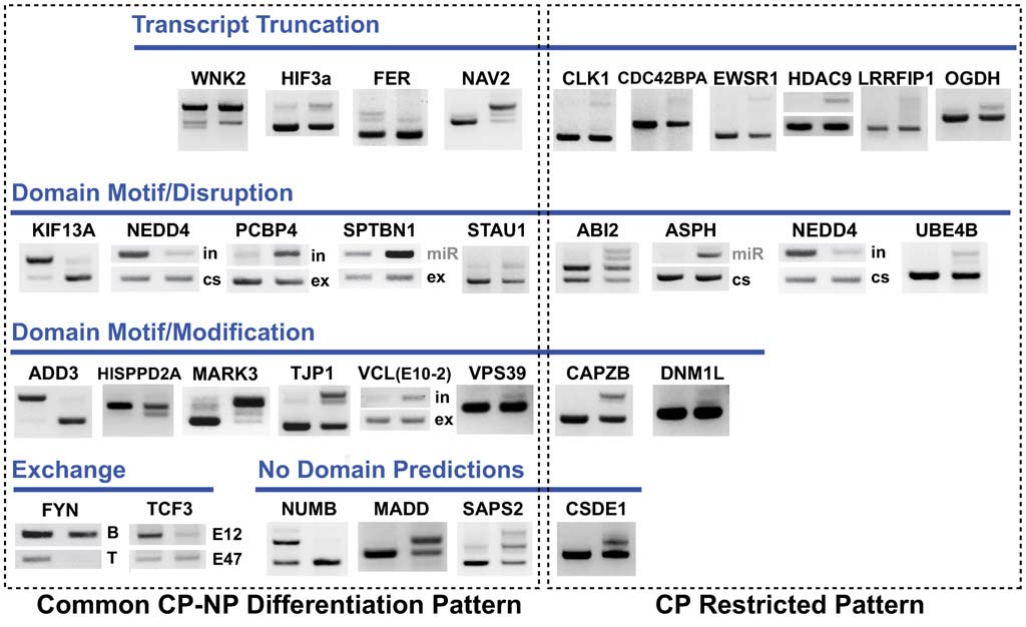
Genes with confirmed AS events have distinct domain-level changes. RT-PCR results for a panel of predicted CP differentiation-splicing events with both a common CP-NP differentiation and CP-specific ANOVA pattern. Genes are categorized based on predicted domain/motif changes: truncation, disruption, modification, exchange or no associated predictions. The higher band in each gel image is the longer isoform with exon inclusion (in); the lower band is the shorter isoform with exon exclusion (ex), unless indicated as a constitutive (cs), mutually exclusive (mx), or miRNA (miR)-containing exon. Additional confirmed genes are shown in Figures 3 and 7 and are further described in Table 3.

Fifty alternative exons with a CP-specific or common CP-NP differentiation pattern were selected for further confirmation and in-depth analysis of domain/miRNA binding sites. When applied to a previously described dataset with comprehensive validation (knockdown of the splicing factor PTB) [45], AltAnalyze identified a high percentage of true-positive splicing events (Text S2). To better assess the impact of splicing on predicted domain/miRNA binding site composition, genes containing such predictions with few alternative exons were preferentially tested. RT-PCR analysis confirmed 44 of the 50 target alternative exons, with 37 displaying significantly larger shifts in isoform expression than the rest (Figure 5 and Figure S2). The six failed primer sets produced either inconclusive results or missing PCR products. For all genes except VCL, only one alternative exon per gene was tested, even if multiple exons were predicted.

**Figure 6:**
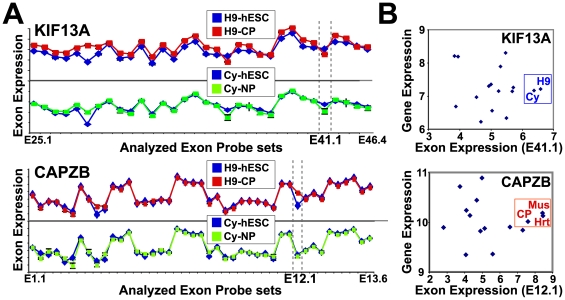
Comparison of differentiation and tissue AS patterns. (A) For the genes KIF13A and CAPZB, log2 expression values for exon aligning probe sets are shown; probe sets are ranked in order of genomic position on the *x*-axis and expression values are plotted on the *y*-axis. (B) For both genes, relative exon-inclusion is assessed for CP and NP differentiation conditions and 11 adult tissue conditions by plotting the mean constitutive gene expression (*y*-axis) against the expression of the interrogated alternative exon (*x*-axis). Each diamond represents a distinct tissue. For KIF13A, exon 41 (E41) is most highly expressed in the hESC lines (H9 and Cythera), suggesting E41 inclusion is greatest in hESCs. For CAPZB, exon 12 (E12) is most highly expressed in muscle (CP, heart and muscle). H9 = REX+ hESC, Cy = Cythera hESCs, Mus = muscle, Hrt = heart.

Genes with some of the most pronounced confirmed changes and a common CP-NP differentiation AS pattern included those encoding serine/threonine kinases (SLK, FER, FYN, MARK3), spectrin-actin binding (SPTBN1, ADD3), cell cycle (MADD, PCBP4, SEPT6), and cell-cell communication (TJP1) proteins. Similarly regulated genes with a CP-specific AS pattern included those encoding calcium signaling (ASPH, ANXA7, ATP2A2), cell metabolism (PKM2, OGDH), cell cycle (NUMB, UBE4B, CSDE1, NF1, ANXA7), and double-stranded RNA binding (STAU1) proteins. Several of these confirmed AS events appeared to have cardiac/muscle-specific and common CP-NP differentiation patterns when examined with the entire adult tissue/cell line exon-array panel. This was the case for the genes KIF13A and CSDE1, each of which showed the highest alternative exon expression for hESCs or cardiac/muscle cells, respectively, when compared to all other tissues and cells (Figure 6A–B). Thus, most of the examined alternative exons have expression patterns consistent with those predicted by AltAnalyze and appear to mimic those in adult tissues.

### Identifying Splicing Events Predicted to Modify Protein Function

The possible effects of AS on protein function are diverse and therefore challenging to predict bioinformatically. Since AltAnalyze identified confirmed domain/motif changes that correlate with changes in protein function (e.g., PKM2 [41],[42] and VCL [43]), we chose to further explore AltAnalyze's predictions for the 44 confirmed alternative exons. These analyses are useful for evaluating how splicing may impact protein domain/motif inclusion and composition for future validation studies.

### High Degree of Specificity of Domain-Level Protein Predictions

Of the 44 confirmed splicing events for CP differentiation, 34 were initially predicted to alter protein domain or motif composition (Table 3). Although validation of these events requires careful *in vitro* analyses, to evaluate the specificity of AltAnalyze protein predictions, we examined the genomic overlap of an alternative exon with domains/motifs (direct genomic alignment) and performed a more exhaustive competitive protein analysis to determine whether any comparisons yield an absence of changes in domain/motif composition (Text S1). This exhaustive method examines all possible competitive protein isoform pairs and selects the pair that produces the smallest overall differences in protein sequence and domain/motif predictions.

**Table 3:** AltAnalyze functional predictions for confirmed CP differentiation AS events.

Gene symbol	ANOVA pattern	SI	Exon ID	GE fold Δ	Exon annotations	Δ protein length	Primary functional change
CSDE1	cardiac	−4.01	E3	0.05	cassette exon	767->767	
MADD	diff	−2.04	E28	0.24	cassette exon	1608->1581	
NF1	cardiac	−2.28	E57	1.07	cassette exon	2127->2145	
NUMB	diff	1.59	E14	−0.1	cassette exon	651->603	
SAPS2	diff	−2.27	E3|E4	0.17	cassette exon	932->966	
**Transcript Truncation**							
CDC42	diff	−1.94	E9-1	0.25	alt-C-term	116->191	miRNA (gain), GTPase Rab/Ras/Rho (gain)
CDC42BPA	cardiac	−1.8	E37	0.28	cassette exon	1719->1045	Kinase (loss), PAK box Rho BIND (gain/loss)
CLK1	cardiac	−2.83	I7	0.4	intron-retention	454->134	Kinase, H+ acceptor (loss)
EWSR1	cardiac	−2.03	E16-2	−0.3	intron-retention	600->146	DNA BIND, RRM (loss)
HDAC9	cardiac	−3.12	E4	0.78	cassette exon	1066->21	Interaction w/MEF2, HDAC region (loss)
HIF3A	diff	−1.67	E8-3	0.06	Alt-5′	237->363	DNA BIND, HLH, PAS, Nuc_translocat (gain)
LRRFIP1	cardiac	−3.34	E5 to E9	0.68	cassette exon	752->640	DNA BIND, PT, PS (loss)
NAV2	diff	−2.96	E21|E22	0.77	cassette exon	1493->2429	Calponin_act_bd, Na_channel4 (gain)
OGDH	cardiac	−3.14	E6	−0.04	cassette exon	1023->567	2 oxoglutarate_DH_E1, Transketo_Cen_R (loss)
WNK2	diff	−2.16	E28	−0.28	cassette exon	45->1004	Kinase, H+ acceptor, PS (gain)
FER	diff	1.47	E4	1.54	cassette exon	163->822	Kinase, H+ acceptor, SH2 (gain)
**Disrupted Domains/Motifs Sequences**							
ABI2	cardiac	−1.8	E9	−0.19	cassette exon	401->513	Neu_cyt_fact_2 (gain)
ANXA7	cardiac	−4.04	E6	−0.32	cassette exon	466->488	Annexin (gain/loss), Pro-rich (loss)
ASPH	cardiac	−1.66	E7|E8	0.96	cassette exon	313->225	miRNA, Asp-b-hydro N-term (gain/loss), Cytoplasmic/Lumenal Topo, PS (loss)
ATP2A2	cardiac	1.42	E20|E22	1.38	bleedingExon	1042->997	miRNA, Cytoplasmic Topo (loss)
KIF13A	diff	2.03	E41	1	cassette exon	1805->1770	PS (loss)
NEDD4	diff	1.12	E7	−0.02	cassette exon	1000->1247	C2 Domain (loss)
PCBP4	diff	1.35	E6-3	0.39	intron-retention	369->397	KH 1 (loss)
SPTBN1	diff	−1.96	E34-2	0.45	bleedingExon	2377->2155	miRNA (gain), Carbohyd-O-linked, Spectrin, PH (loss)
STAU1	diff	−1.52	E7	−0.04	cassette exon	496->577	dsRNA BIND (gain)
UBE4B	cardiac	−1.97	E9	−0.41	cassette exon	1173->1302	Phosphopantetheine attachment (loss)
**Exchanged Domain/Motif Sequences**							
FYN	diff	1.57	E12	−0.1	cassette exon	534->482	Kinase (gain/loss)
PKM2	diff	−2.64	E12	0.32	cassette exon	531->531	Kinase (gain/loss), ISC, FBP, PT (loss)
TCF3	diff	−1.33	E18	−0.75	cassette exon	654->651	AnnexinVII (loss), bHLH (gain/loss)
**Modified Domain/Motif Sequences**							
ADD3	diff	2	E16	0.26	cassette exon	706->674	Oxred_Ald_Fedxn_C-term (gain/loss)
CAPZB	cardiac	−3.77	E12	−0.21	cassette exon	272->277	Factin_cap_beta (gain/loss)
DNM1L	cardiac	−2.18	E3	−0.1	cassette exon	736->751	Dynamin GTPase (gain/loss)
HISPPD2A	diff	1.68	E49-1	−0.17	alt-5′|cassette exon	1433->1412	HisAc_phsphtse (gain/loss)
MARK3	diff	−1.52	E19	0.04	cassette exon	729->744	Kinase (gain/loss)
SLK	diff	2.44	E15	0.64	cassette exon	1235->1204	Kinase like (gain/loss)
TJP1	diff	−2.27	E24	−0.03	cassette exon	1676->1748	ZU5 Domain (gain/loss)
VCL	diff	−1.08	E23	−0.1	cassette exon	1066->1134	Vinculin/catenin (gain/loss)
VCL	Diff	−1.64	E10-2	−0.1	alt-5′	1066->222	Vinculin/catenin, PS, PT (loss)
VPS39	Diff	−1.53	E3	0.08	cassette exon	875->886	Citron homology, WD40 (gain/loss)
**miR binding site(s)**							
C6orf134	Cardiac	−1.37	E11	0.19	alt-C-term	398->300	miRNA (gain)
DERP6	Diff	−1.68	I8	−0.45	intron-retention	316->279	miRNA (gain)
LEFTY1	Diff	1.19	E4	−0.68		366->366	miRNA (loss)
MAFB	Diff	−1.01	E1-5	0.41		323->323	miRNA (gain)
SEPT6	Diff	2.4	E11	0.59	cassette exon	427->429	miRNA (loss)

Splicing, protein, and miRNA binding site annotations are shown for alternative exons confirmed by RT-PCR. For each alternative exon, the corresponding gene name (Gene symbol), ANOVA AS differentiation pattern (ANOVA pattern: diff = common CP-NP differentiation, cardiac = CP-specific), splicing index (SI) fold change, relative AltAnalyze exon/intron position in the gene structure (Exon ID), gene-expression (GE) fold change (Δ) for the gene, AS annotations that correspond to the Exon ID, change in predicted protein length (length of the competitive protein isoforms in hESC->CP), and top corresponding domain/motif or miRNA binding site annotations (Primary functional Δ). Negative SI fold changes indicate increased alternative exon expression in CPs and vise versa. For primary function Δ annotations, gain indicates the increase in the expression of an alternative exon overlapping with that domain in CPs versus hESCs, a loss indicates a relative decrease in expression and a gain/loss indicates that the domain/motif is present in both protein isoforms but with different sequence. PS = phosphoserine modified residue, PT = phosphotyrosine modified residue, miRNA = miRNA binding site. Complete annotations can be found in Dataset S2.

Twenty-two of the 34 alternative exons were predicted to have domain/motif changes with both direct genomic alignment and competitive isoform analysis. These alternative exons should directly change the sequence or disrupt a domain/motif and thus represent higher-confidence predictions. Only one gene, LEFTY1, was predicted to alter the sequence of a domain (transforming growth factor β) with direct genomic alignment and not the competitive isoform analysis. In all but four of these 34 alternative exons, changes in domain/motif composition were also predicted by the exhaustive comparison method. Three of these four alternative exons were present in both untranslated and coding regions of the different possible isoforms. Since the exhaustive method is biased towards selection of competitive isoforms that produce no change in domain/motif composition, only competitive isoforms where the alternative exon was present in an untranslated region were selected. Of the remaining 30 alternative exons, 17 had identical domain/motif predictions with the exhaustive and the original competitive isoform analysis and 13 had almost identical predictions (largely the same but sometimes fewer domain/motif changes) with the exhaustive method (Dataset S2). Thus, several of the domain/motif changes were only found with the competitive isoform analysis and not with direct genomic alignment or the exhaustive methods. However, it is unclear which predictions are false positives, since AS of a single exon could theoretically co-segregate with other AS or AP events in the same transcript.

### Impact of Domain-Level Predictions on Known Biology

Predicted changes in protein domain/motif composition for confirmed splice events could be classified into four groups: truncation, disruption, exchange, and modification (Figure 5). For protein truncation or disruption, we can infer potential functional consequences of the domain/motif change based on known biology of the protein and its interactions. For exchange or modification, we can only speculate that the protein function differs from that of the characterized isoform(s).

### Protein Truncation with CP Differentiation

Nine of the confirmed AS events (CDC42, CLK1, EWSR1, FER, HDAC9, LRRFIP1, OGDH, VCL (exon 10-2), and WNK2) were predicted to introduce a premature stop-codon into the transcript, causing either protein truncation (>30% decrease in protein length) or absence of translation (e.g., nonsense-mediated decay) [6]. In the majority of cases, except for FER and LRRFIP1, no protein sequence was found in public databases for the truncated isoform; therefore, AltAnalyze produced theoretical protein sequences based on *in silico* translation. In four cases (WNK2, CLK1, HDAC9, EWSR1), the *in silico* predicted protein was 2–30% of the length of the competitive isoform and thus likely not to be translated. In addition to these C-terminally truncated proteins, N-terminal truncation of CDC42BPA, HIF3A and NAV2 resulted in substantially shorter protein sequences (35–39%) with a resulting loss in domain/motif predictions. There did not appear to be a bias towards increased protein truncation in CPs or hESCs.

Since these splicing events are predicted to significantly reduce protein size and domain/motif composition, there is a much higher likelihood that these changes would disrupt protein function or prevent protein translation. For example, in cardiomyocytes, the large upregulation (∼8-fold by AltAnalyze) of a cassette exon in the histone deacetylase HDAC9 protein is predicted to truncate the reference isoform from 1066 to 21 aa. HDAC9 typically represses expression of myocyte enhancer factor 2 (MEF2), a potent cardiac inducing transcription factor [46]. Truncation or more likely absence of translation should thus alleviate HDAC9's repressive action on MEF2 transcription factors to promote cardiogenesis. Among the four MEF2 family members examined in this analysis, three were transcriptionally upregulated, with MEF2C increased 51-fold in CPs versus ESCs. In the case of hypoxia-inducible factor-3α (HIF3A) (common CP-NP differentiation pattern), the putative truncated isoform (lower band by RT-PCR) was expressed in both hESCs and CPs, while the full-length isoform was largely restricted to CPs. The truncated form of HIF3A, resulting from selection of an alternative 5′ splice site, is predicted to disrupt the DNA-binding, PAS, and nuclear translocation domains and the helix-loop-helix motif. HIF3A participates in the adaptive response to hypoxia as a transcriptional regulator of downstream genes [47]. The precise function of this variant is unclear, but its exon and domain structure are similar to those of a mouse variant of this gene called inhibitory PAS domain protein, a dominant-negative regulator of HIF3A transcription [47]. Thus, splicing of HIF3A may play a critical role in regulating hypoxic responses in pluripotent versus differentiated cells.

### Disruption of Domains and Motifs with CP Differentiation

In addition to protein truncation, removal of protein sequences was also predicted to disrupt domains and motifs in 10 of the confirmed AS events (ABI2, ANXA7, ASPH, ATP2A2, KIF13A, NEDD4, PCBP4, SPTBN1, STAU1, and UBE4B). In CPs, these predictions include the disruption of the C2 calcium-dependent membrane targeting domain in the NEDD4 protein with exclusion of a 72-aa block of exons; intron retention in the PCBP4 gene, which produces a shorter N-terminus that disrupts a KH domain; and the disruption of a phosphopantetheine attachment site in the UBE4B protein with the insertion of a cassette exon encoding 129 aa. In hESCs, the disruption of presumptive domains was observed with the exclusion of 61-aa-encoding exon in the ABI2 protein that eliminated the predicted presence of a neutrophil cytosol factor domain; and removal of the first 9 aa from the double-stranded DNA binding domain in the STAU1 gene.

Since these domains are crucial for the annotated functions of these genes, the predicted sequence loss or disruption could affect their function considerably. An example is PCBP4, an RNA-binding protein and regulator of apoptosis characterized by presence of a KH domain. PCBP4 with an intact KH domain can suppress cell proliferation by inducing apoptosis, but is largely absent in hESCs. Since PCBP4 has a common CP-NP differentiation-splicing pattern, AS of this gene may be important in maintaining pluripotency in hESCs.

Two other, genes aspartyl beta-hydroxylase (ASPH) and spectrin, beta, non-erythrocytic 1 (SPTBN1) both had prior evidence of functionally distinct splice variants, linked in this case to the regulation of cardiac physiology. In the case of ASPH, the cardiac-enriched form specifically complexes with cardiac contractile components (calsequenstrin, triadin, and the ryanodine receptor) [48] in the release of sarcoplasmic calcium; in contrast, the hESC-enriched form is highly expressed in neoplastic cells, has a distinct cellular localization, and has an additional enzymatic domain that regulates growth factor activity [49],[50]. Proteins encoded by SPTBN1 are found in the sarcomere along the muscle Z-line and likely contribute to structural stability [51]. Consistent with AltAnalyze predictions, upregulation of a short form of SPTBN1 in CPs and NPs is associated with the loss of the pleckstrin homology domain (producing a loss of inositol-1,4,5 triphosphate binding) [52] and when associated with spectrin alpha 2, the shorter protein forms more stable tetramers than the longer protein [53]. Other splicing events had less clear functional implications on protein sequence, such as the microtubule-dependent motor protein KIF13A, in which removal of an exon encoding 35 aa results in the loss of one of three phosphoserine sites indicated by UniProt. If modulated directly by a protein kinase, however, such a change could alter the regulation of the resulting protein.

### Exchange of Domain Sequences by Mutually Exclusive AS

Unlike the disruption of a critical protein domain, the functional impact on a domain with an altered sequence is less clear. As shown in the case of PKM2, mutual-exclusive splicing can alter the presence of functionally important protein residues without significant changes in overall protein sequence. This was also the case for the E2A immunoglobulin enhancer-binding factor TCF3 and for the serine/threonine and protein-tyrosine kinase FYN, in which a DNA-binding or kinase domain is specifically altered by the mutually exclusive exchange of a cassette exon of similar lengths, respectively. Interestingly, the mutually exclusive isoforms of TCF3 and the FYN have different biochemical properties [54],[55], suggesting that the domain-level alterations predicted by AltAnalyze correlate with function. In the case of TCF3, although the DNA-binding domain is 76% identical between the mutually exclusive isoforms, the hESC-enriched isoform (E12) has less DNA-binding affinity than the differentiation-enriched form (E47). Thus, AS of mutually exclusive exons is a potent means to modify specific residues within a sequence block without significantly changing overall protein length.

### Modification of Domain/Motif Composition with AS

Although some confirmed AS events significantly changed a domain sequence, the domain was still called present in both alternative isoforms. This was the case for nine genes with confirmed alternative exons (ADD3, CAPZB, DNM1L, HISPPD2A, MARK3, SLK, TJP1, VCL, and VPS39). Specific examples include the removal of 32 aa in the C-terminal aldehyde ferredoxin oxidoreductase domain of the ADD3 protein, insertion of 13 aa into the dynamin GTPase region of DNM1L, modification of the C-terminal end of the F-actin capping beta subunit region of CAPZB, and removal of 11 aa from the N-terminal Citron homology domain (CNH) of VPS39. In each case, except VSP39, altering the sequence has unknown consequences for protein function. VPS39 is a putative adaptor protein that displays downregulation of a cassette exon in hESCs relative to CPs. The CNH domain in this protein is required for the clustering and fusion of late endosomes and lysosomes [56]. Interestingly, the TRAP-1 homologue, the isoform that lacks this exon, does not mediate lysosomal clustering. Rather, it specifically associates with the transforming growth factor β signaling pathway, suggesting that modification of the CNH domain can alter its signaling properties.

### Missing Domain Annotations Affect the Sensitivity of AltAnalyze Prediction

At least two confirmed AS events (NUMB and MADD) had no difference in domain-level predictions, but did have known functional isoform differences associated with the AS events [57]. NUMB or the *Drosophila* orthologue NUMB is involved in early cell-fate decisions [57] and MADD (MAP-kinase activating death domain) protein is a membrane-bound cytoplasmic adaptor protein that interacts with the TNF-α receptor 1 to transduce apoptotic signals [58]. Both genes affect pathways of proliferation and apoptosis. The CP-enriched isoform of NUMB is antiproliferative, whereas the hESC-enriched form (p71), with a longer proline-rich region (PRR), retains its proliferative properties [59],[60]. Likewise, expression of the CP-enriched MADD isoform (IG20) promotes apoptosis, whereas the hESC-enriched isoform (DENN) is anti-apoptotic and is typically over-expressed in tumors. These missing annotations were likely due to either an absence of the domain annotation (PRR) or lack of an annotated domain/motif. These examples illustrate the dependence of AltAnalyze's domain/motif predictions on up-to-date and valid annotations.

### Developmental Regulation of miRNA Binding Site Inclusion by AS

**Figure 7:**
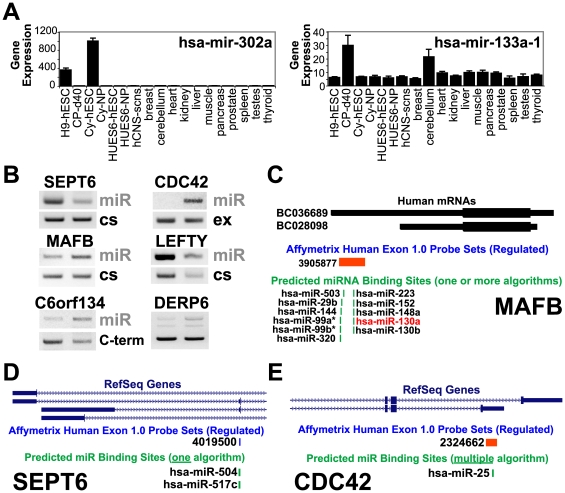
Both miRNAs and miRNA binding sites are regulated with hESC differentiation. (A) Expression profiles of two previously characterized miRNAs, mir-302a and mir-133-1, from combined tissue/cell-line gene expression data. (B) RT-PCR isoform expression of genes with putative miRNA binding sites within the regulated probe set. The presence of one or more putative miRNAs is indicated by the notation miR. (C–E) The 3′ region of genes corresponding to three genes are shown, where the regulated isoforms are displayed from the UCSC genome browser along with regulated probe sets and putative miRNA binding site locations. Exons are indicated by thin boxes, UTR regions by thinner boxes and introns by a line with overlapping arrows. Each gene (MAFB, SEPT6, and CDC42) represents distinct possible modes of exon regulation that lead to altered miRNA binding site inclusion: shorter 3′UTR, alternate cassette exon inclusion, and alternate C-terminal exon. Both MAFB and SETP6 are on the reverse genomic strand, where orientation is 3′ to 5′. The term ”multiple algorithms” indicates that two or more miRNA binding site prediction algorithms (PicTar, miRanda, miRbase or TargetScan) predicted a binding site in aligning probe sets.

A number of recent studies demonstrated a critical connection between miRNA expression and the maintenance of pluripotency or the differentiation of cardiac cells from hESCs. In our exon-array gene expression analysis, genes for 26 miRNAs were up- or downregulated during differentiation to CPs and NPs, including previously implicated pluripotency (mir-302a, 302b) [61] and cardiac (mir-133, 23b, 26a) [44] regulated miRNAs (Dataset S1). Each of these miRNAs was appropriately segregated by ANOVA pattern analysis (Figure 7A).

Although much effort has been devoted to defining the expression patterns and novel targets of miRNAs, little is known about the role of AS in miRNA binding site inclusion in processed mRNA transcripts. Traditional gene expression microarrays focus on the coding regions of transcripts and ignore the noncoding exons, which can be alternatively spliced to produce different C-terminal exons or 3′UTRs of different lengths. However, exon-tiling arrays allow us to assess mRNA features in tandem with existing predictions for miRNA binding site position on a global basis.

Our analysis identified 264 genes with putative miRNA binding sites that overlap with alternative exons, including those undergoing AS. We tested 10 of these alternative exons by RT-PCR and confirmed nine, including the SPTBN1 and ASPH variants described earlier. Putative miRNA binding sites were included or excluded as a result of alternative cassette exons (ASPH, SEPT6), alternative C-terminal exons (CDC42, C6orf134), bleeding exons (SPTBN1), intron retention (ATP2A2, DERP6), or 3′UTRs with a longer or shorter sequence (LEFTY1, MAFB) (Figure 7B–D). At least one of these alternative exons (MAFB), with predicted regulation of a mir-130a binding site, is a known target of the predicted miRNA [62] (Figure 7C). In addition to MAFB, several of the putative regulated binding sites were independently predicted by multiple miRNA binding site algorithms (ATP2A2, C6orf134, CDC42, LEFTY1).

Examination of miRNAs with previously established hESC or cardiac differentiation expression patterns identified binding sites for mir-302a, 302c (ESC), and mir-26a (cardiac) in the alternative bleeding exon of SPTBN1 and the afore mentioned binding sites in the 3′UTR of ATP2A2. These data suggests a new, largely AS-dependent mechanism for miRNA regulation of such genes. Since miRNAs can promote and inhibit the translation of targets dependent on cell cycle stage [63], there is the opportunity for complex modes of regulation by these predicted targets *in vivo*. Future studies will be aided by a global profile of miRNAs expression in hESCs and CPs, to determine which miRNAs are most likely to target these alternative mRNAs.

### Summary and Conclusions

Using high-density expression profiling, a new method for isolating cardiomyocytes, and novel bioinformatics methods (AltAnalyze), we characterized AS along distinct developmental pathways that influence both pluripotency and the commitment to cardiac and neural lineages. In addition to new insights into these processes, these results offer novel targets for driving the expression of pluripotent cells to distinct lineages and inducing pluripotency from adult cells at the level of specific splice isoforms.

We identified genes that undergo AS during differentiation and observed several global trends which suggest that functional elements, such as protein domains and miRNA binding sites, are coordinately regulated by AS. Many alternative exons highlighted in our analysis were predicted to disrupt or modify functionally important sequences, such as DNA-binding and protein kinase domains that are likely impact protein function. Several of our domain-level predictions also correlated with known changes in protein isoform function or expression as a result of AS [36], [41]–[43]. Thus, AltAnalyze may be useful for identifying AS events that alter protein function/expression by disrupting or modifying protein domains, motifs, or miRNA binding sites.

We identified and confirmed many splicing events that occurred along pathways of apoptosis and proliferation. Two genes confirmed by RT-PCR encode the apoptosis activators PCBP4 and MADD. Isoforms for both genes that induce apoptosis, were downregulated in hESCs but not CPs. Conversely, the proliferation-promoting isoform of NUMB is expressed in hESCs but is undetectable in CPs, while the anti-proliferation isoform is upregulated in CPs, as shown by RT-PCR. These results suggest the intriguing possibility that splicing may coordinately alter the functional repertoire of distinct members of the same pathway to elicit a biological effect, in this case, self-renewal in hESCs. We also observed AS of the apoptotic regulators CSDE1 and UBE4B along with previously demonstrated tumor suppressor genes ANXA7 [40], EWSR1 [64], and PKM2 [41]. Since both PKM2 and the proto-oncogene EWSR1 directly interact with the pluripotency transcription factor OCT3/4 to promote OCT3/4 activity [41],[64], specific isoforms of these genes may also be critical in the regulation of hESC maintenance.

Although only one confirmed CP-specific AS event (ASPH) was clearly linked to the regulation of cardiac physiology, several other novel CP-specific AS events were predicted to alter the composition of critical protein domains (CAPZB, UBE4B, HIF3A, HDAC9). One of the most intriguing was AS of the cardiac inhibitor HDAC9, which results in a highly truncated or nonexpressed form specifically in CPs. These data further support a role for AS in the direct specification of cardiac precursors.

Finally, analysis of the overlap between predicted miRNA binding sites and alternative exons revealed a potential mechanism by which specific cell types may regulate miRNA activity independently of miRNA expression. Such regulation involves AS of exons and differential expression of distal terminal exons, where the mechanism regulating exon length is unclear. Two recent analyses have further demonstrated the interaction between miRNAs and alternatively spliced isoforms [65] or UTRs of different length [34]. Since miRNA expression is thought to fine-tune protein expression downstream of transcription, alternative exon inclusion may be a parallel means of regulating miRNA binding site selection while still retaining full-length protein expression.

While we present several new analyses in this study, it will be essential to experimentally validate these protein and miRNA-level predictions. Additional computational analyses, such as comparative genomic sequence analysis, will also be important for delineating common and distinct cis-regulatory sequences that may regulate cardiac and neuronal splicing. Further refinement of our algorithm to decrease false negatives, similar to other approaches [45],[66], will also be important in identifying additional AS events. Finally, future application and refinement of these analyses to additional cell lineages and time points may yield greater resolution of AS events that will likely provide insights into the mechanisms of cell-fate commitment and maintenance of hESC pluripotency.

## Supporting Information

Figure S1 Segregation of transcriptional profiles by comparison of neural and cardiac differentiation. Patterns of gene expression are shown for two analyzed pattern groups, (A) common to neural and cardiac differentiation or (B) specific to CPs. Adjacent to each heatmap are the top-ranked genes based on ANOVA p values for each specific pattern; genes highlighted in blue are associated with ESCs or self-renewal, and genes in red with cardiac-specification. Gene Ontology (GO) terms and pathways enriched in the (C) common or (D) cardiac-specific differentiation pattern groups are displayed as compared to the number of associated gene changes in each of the two pattern groups. Asterisks indicate significant GO-Elite scores (permute p<0.01) in the alternate pattern group.
(0.55 MB EPS)

Figure S2 The first column in each gel is for RNA from REX+ hESCs and the second is CPs. The numbers listed under these columns are the predicted amplicon lengths for those reactions. Left adjacent tick marks indicate predicted amplicon positions. Mx-mx  =  mutual-exclusive splicing, bleeding  =  exon bleeding, miR = miRNA binding site (predicted), ex  =  exon exclusion isoform, in  =  exon exclusion isoform, cs  =  constitutive mRNA region.
(3.97 MB EPS)

Table S1 Primer sequences for confirmed and non-confirmed AS events.
(0.02 MB PDF)

Text S1 Supplemental methods file. Includes detailed descriptions of algorithms, expression filtering and database architecture of AltAnalyze.
(0.35 MB DOC)

Text S2 Analysis of sensitivity and specificity of AltAnalyze predictions with a publicly available alternative splicing validation dataset (PTB knockdown).
(0.07 MB DOC)

Dataset S1 Gene expression results from the human exon array analysis for all conditions examined. Gene annotations, statistics, ANOVA patterns and log2 expression values provided for all Ensembl genes.
(9.48 MB ZIP)

Dataset S2 Alternative exon results for CP differentiation. Multiple spreadsheets are included. Complete probeset- and gene-level results along with microRNA binding site and protein domain/motif over-representation analysis results from AltAnalyze are provided. Additional ANOVA pattern, splicing calls and cross-tissue comparison information is included.
(5.20 MB ZIP)

Dataset S3 Alternative exon results for NP differentiation. Multiple spreadsheets are included. Complete probeset- and gene-level results along with microRNA binding site and protein domain/motif over-representation analysis results from AltAnalyze are provided.
(5.18 MB XLS)

Dataset S4 Gene Ontology and pathway over-representation analyses. The top-scoring terms from GO-Elite are provided for all comparisons.
(0.17 MB XLS)
