# Supplementary material for: Alternative Splicing in the Differentiation of Human Embryonic Stem Cells into Cardiac Precursors
Source: PLoS Comput Biol. 2009 Nov 6;5(11):e1000553. doi: 10.1371/journal.pcbi.1000553 (PMC2764345; doi:10.1371/journal.pcbi.1000553)
Supplement: Table S1 — Primer sequences for confirmed and non-confirmed AS events. (0.02 MB PDF) [file pcbi.1000553.s003.pdf]

Confirmed

Primers	Sequence
L-CSDE1_2-4_Flank	AAAGTGCAATATTTGAGTATCACTGC
R-CSDE1_2-4_Flank	TTCACCAGTTTAACAGCAATGG
L-MADD_25-27_Flank	CCAGCGAGTCTATCTCTATGAGG
R-MADD_25-27_Flank	GAGGTTGTGCAGAAGTGTGG
L-NF1_48-50_Flank	TGACCTCACTTGCAACTTCC
R-NF1_48-50_Flank	AAGTGCAGCATTACAACATGG
L-NUMB_7-10_Flank	TGGCACCACAATCTCCTACC
R-NUMB_7-10_Flank	TCCCTTGGAAAGGTGATGC
L-SAPS2_1-4_Flank	CCACAAATGTTTTTCTGTTTTGC
R-SAPS2_1-4_Flank	AGCAGCTTCTGGTTCTGAGC
L-CDC42_5-7_Int5'	TGCCAAGAACAAACAGAAGC
R-CDC42_5-7_Int5'	GAGGCTTTCAAACAGGATGG
L-CDC42_5-7_Junc3'	AAGAAAAGTGGGTGCCTGAG
R-CDC42_5-7_Junc3'	ACATTCTTTAGGCCTTTCTGTG
L-CDC42BPA_33-35_Flank	ACATGGGTCCTGGAGATGG
R-CDC42BPA_33-35_Flank	ATCACTTCCTTGGTCCATGC
L-CLK1_7-8_Flank	GACTGGATCATATCAGAAAGATGG
R-CLK1_7-8_Flank	TCGTCATCATATGTTGCACTACC
L-EWSR1_14-18_Flank	ACTGGCAGTGTCCCAATCC
R-EWSR1_14-18_Flank	CCCATCTGTTCCATCAAAGG
L-HDAC9_2-4_Flank	TTGGCTCAGCAAAGAATGC
R-HDAC9_2-4_Flank	GCCGTGTCAAGTTCTCATGC
R-HIF3A_6-8_Flank	CAGACGATACTCTCCGACTGG
L-HIF3A_6-8_Flank	ACCTGGAAGGTGCTGAACTG
L-LRRFIP1_2-10_Flank	GCGAGATCCGCATGAAGG
R-LRRFIP1_2-10_Flank	TTCCTTGATTTCCCTGATGG
L-NAV2_15-18_Flank	TGTCAGAACTAACAGTGTGAAGACC
R-NAV2_15-18_Flank	GGATCATACTGCCCATCAGC
L-OGDH_4-6_Flank	AGCCCAACGTGGACAAGC
R-OGDH_4-6_Flank	GATGATCTCCCGCAGAGG
L-WNK2_23-27_Flank	GCAGGCAAGCTGCTAAATCC
R-WNK2_23-27_Flank	CCATGTCTTGCAGCTTCTGG
L-FER_2-4_Flank	ACACCTCATCATGGACACG
R-FER_2-4_Flank	GGCCATAAATTTCTTTACTGTTTCC
L-ABI2_2-4_Flank	TCTCCTCCCAGTGTCTTTCC
R-ABI2_2-4_Flank	ACTCAACCACAGCAGCTTCC
L-ANXA7_5-7_Flank	TCAGTATCCTGGAGGACAACC
R-ANXA7_5-7_Flank	AAACCCTTCATTGCCTTACG
R-ASPH_6_cterm1	TCCTTCCTGGATAGGTCTGC
R-ASPH_7_cterm2	TTGTTGTGGTTCTCCTGTGG
L-ATP2A2_20-23_Internal5'	CCTCTATGTCGAACCCTTGC
R-ATP2A2_20-23_Internal5'	CACCAGGGGCATTATGAGC
L-ATP2A2_20-23_Junction5'	CCTGGAACCTGCAATACTGG
R-ATP2A2_20-23_Junction5'	CCTCTTGCAGCAAAGAAACC
L-KIF13A_37-39_Flank	AAAAGGATAGCCCTGGAAGC
R-KIF13A_37-39_Flank	GGACACTTCTTTCTCCAAGACC
L-NEDD4_1-3_Flank	AAGAGACAGCAACAGAGATTGC

R-NEDD4_1-5_Flank	TGAGGGGTTGGTCTTTTCC
L-NEDD4_Const5'	TTGCAGCAACAACAAGAACC
R-NEDD4_Const5'	GGCCTGGTTGCTATACATGG
L-PCBP4_3-6_Flank	GAAGGGCGAGACTGTAAAGC
R-PCBP4_3-6_Flank	AGCCTTCCCAATCAGTGAGC
L-SPTBN1_31-34_Int5'	GAGCACGAAGGTTTCAGAGG
R-SPTBN1_31-34_Int5'	ACCCAGAGGATTTGGAAAGG
L-SPTBN1_31-34_Junc5'	AGGGATCTCCACGGGATG
R-SPTBN1_31-34_Junc5'	ACTCGTGTTTCCGATTGAGG
L-STAU1_2-4_Flank	TTCGTCCCTTCTTCCTCTCC
R-STAU1_2-4_Flank	GCAGCCTGTCTTGTCTTTCC
L-UBE4B_6-8_Flank	AACAAGCCCCATGTTCTGC
R-UBE4B_6-8_Flank	TCAGAAGCTGGCTGACTGC
L-FYN_6_Internal5'	TTATCCGCGAGAGTGAAACC
R-FYN_8_Mx1-Incl	GACAGATCGGTAAGCCTTGG
R-FYN_9_Mx1-Incl	TTCTCCAGACACAACGAACG
L-PKM2_8-10_Excl-Incl	TGGAGAGCATGATCAAGAAGC
R-PKM2_8-9_Internal5'	GGTCTGTGGAGTGACTTGAGG
R-PKM2_8-10_Excl	CCACTGCAGCACTTGAAGG
L-TCF3_16	CTCGGAGGAGGAGAAGAAGG
R-TCF3_16-18_Mx1-Incl	GATGAGCAGCTTGGTCTGC
R-TCF3_16-19_Mx2-Incl	GCTCCAAGTTCAGGATGACC
L-ADD3_13-15_Flank	CAATCGAACGTAAACAACAAGG
R-ADD3_13-15_Flank	TCAGGTGACAGGACTTCTTCG
L-CAPZB_8-10_Flank	AAAACAAAGGATATCGTCAATGG
R-CAPZB_8-10_Flank	TGATGCAGCTGTTATGTGACC
L-DNM1L_2-4_Flank	GAAGATAAACGGAAAACAACAGG
R-DNM1L_2-4_Flank	ATCACCTACAGGCACCTTGG
L-HISPPD2A_26_Flank	AGCCATGGCAAAAAGCTACC
R-HISPPD2A_27-29_Flank	TGTGCATGGAATCAAAGAGG
L-MARK3_20-24_Flank	CGAACCGCAACATATAATGG
R-MARK3_20-24_Flank	GCTCATAGTCGCAGTTATTGG
L-SLK_12-14_Flank	GCTGAAGAACCGAAAGAAGG
R-SLK_12-14_Flank	GATCTTTAAGCTGCTGTTTGAGC
L-TJP1_19-21_Flank	AGACTCCCCTGGATTTAAGC
R-TJP1_19-21_Flank	TCCGTATAGCTTGAGGACTCG
L-SEPT6_9-11_Internal5'	GCTGCACGAGAAGTTTGACC
R-SEPT6_9-11_Internal5'	CAGCATGCAGCAAACAGC
L-SEPT6_9-11_Const5'	CGGCACTATGAGCTGTATCG
R-SEPT6_9-11_Const5'	TGGTGCAGTTTCTTCAGACG
L-VCL_21-23_Flank	ATGATGAAGCTCGCAAATGG
R-VCL_21-23_Flank	GTGCTTATGGTTGGGATTCG
L-VCL_6-8_Flank	ACCTCTTGGGATGAAGATGC
R-VCL_6-8_Flank	GCTGCATTTTCCACTTTTGC
L-VCL_6-8_Internal5'	AAACCAAGGCATAGAGGAAGC
R-VCL_6-8_Internal5'	GACAGGTGCCAATCAAAACC
L-VPS39_2-4_Flank	TTGTGGGAACCAAACAAGG
R-VPS39_2-4_Flank	AACAGTGATGCTCCCTTTGC
L-C6orf134_10-12_cterm1	CTGGGAAACTCACCAGAACG

R-C6orf134_10-12_cterm1	AGGATGCAATGGAAGAGTGG
L-C6orf134_1-12_cterm2	AGCAGAGGGAGACATCAAGC
R-C6orf134_1-12_cterm2	AGCTTTGGGCTACCAGACC
L-DERP6/C17orf81_9_Flank	AAGCCAGAGATAGCCTGATCC
R-DERP6/C17orf81_11_Flank	CTCCATAGACAAGGGGAACC
L-LEFTY_4-6_Int5'	CATGATCGTCAGCATCAAGG
R-LEFTY_4-6_Int5'	CAGAGCATTTGTCCATCAGC
L-LEFTY_1_Const5'	AGCCTTCTCAAGGGACAGC
R-LEFTY_1_Const5'	ACCAGCTCCTCCATGTCG
L-ASPH_4_cterm	CTATGATGCTGATGGTGATGG
L-MAFB_6-8_miR-130a	CACTGCACTGAACCAACTGC
R-MAFB_6-8_miR-130a	TTTTTGTCAGTGCAACTTAAATCC
L-MAFB_4-8_Const5'	CATCACCATCATCACCAAGC
R-MAFB_4-8_Const5'	CTGCTGGACGCGTTTATACC

Not-Confirmed

**Primers**	**Sequence**
L-KIF21A_11-13_Flank	GAAGTTGAAAAGAAAAGAAAAGAGG
R-KIF21A_11-13_Flank	CCCCATCAATGTCATCTTCC
L-CTSL1_2-5_Flank	TCACAGTTTAGAGGCACAGTGG
R-CTSL1_2-5_Flank	CTTCATCAGGGCCTTCTCC
L-CTSL1_2-5_Flank	TCACAGTTTAGAGGCACAGTGG
R-CTSL1_2-5_Flank	CTTCATCAGGGCCTTCTCC
L-TRAF6_1-3_Flank	AGCAGAGAAGGCGGAAGC
R-TRAF6_1-3_Flank	AAATGAGCTGGAGAGGTTCC
L-PPP3CB_3-5_Flank	GGCGATTATGTGGACAGAGG
R-PPP3CB_3-5_Flank	GGTTTAAAAGTGCAGCAAGAGG
L-SF3B1_4_Excl_form	GAGCGTCTTGATCCTTTTGC
R-SF3B1_6_Excl_form	CAGCTGTTTGATCCCAACG
L-GAD45B_4-6_Flank	GGAAGAGGAGGAGGAGAAGG
R-GAD45B_4-6_Flank	TGGGTACAGAGCAACTTCAGC
