# Supplementary material for: Alternative Splicing in the Differentiation of Human Embryonic Stem Cells into Cardiac Precursors
Source: PLoS Comput Biol. 2009 Nov 6;5(11):e1000553. doi: 10.1371/journal.pcbi.1000553 (PMC2764345; doi:10.1371/journal.pcbi.1000553)
Supplement: Text S1 — Supplemental methods file. Includes detailed descriptions of algorithms, expression filtering and database architecture of AltAnalyze. (0.35 MB DOC) [file pcbi.1000553.s004.doc]

**Text S1 - Supplemental Methods**

AltAnalyze algorithm and detailed methodologies are provided in this document. This information and additional program documentation can also be found at <http://AltAnalyze.org/help_main.htm>. Referenced source code (.py python files) is provided with the AltAnalyze software as well as through a publicly available SVN repository: <http://code.google.com/p/altanalyze/>.

**Building Probe set to Gene Associations**

Affymetrix Exon 1.0 ST arrays are provided with probe set sequence, transcript cluster, probe set genomic location, and mRNA count annotations from Affymetrix. Each of these annotations are used by AltAnalyze to provide gene, transcript and exon associations. Although transcript clusters represent putative genes, the AltAnalyze pipeline derives new gene associations to Ensembl genes, so that each probe set aligns to a single gene from a single gene database. This annotation schema further allows AltAnalyze to determine which probe sets align to defined exons regions (with external exon annotations), introns, and untranslated regions (UTR).

To begin this process, Ensembl exons (each with a unique ID) and their genomic location and transcript associations are downloaded for the most recent genomic assembly using the AltAnalyze EnsemblSQL.py module, which parses various files on the Ensembl FTP SQL database server to assemble the required fields. This file is saved to the directory “AltDatabase/ensembl/*species*/” with the filename “*species*_Ensembl_transcript-annotations.txt". Since Ensembl transcript associations are typically conservative, transcript associations are further augmented with exon-transcript structure data from the UCSC genome database, from the file “all_mrna.txt” (Downloads/*species*/Annotation database/all_mrna.txt.gz). This file encodes genomic coordinates for exons in each transcript similar to Ensembl. Transcript genomic coordinates and genomic strand data from UCSC is matched to Ensembl gene coordinates to identify genes that specifically overlap with Ensembl genes with the Python program UCSCImport.py. Unique transcripts, with distinct exon structures from Ensembl, are exported to the folder “AltDatabase/ucsc/*species* “*species*_UCSC_transcript_structure_filtered_mrna.txt”, with the same structure as the Ensembl_transcript-annotations file.

Once both transcript-structure files have been saved to the appropriate directory, ExonArrayAffyRules.py calls the program EnsemblImport.py to perform the following steps:

1. Imports these two files, stores exon-transcript associations identify exon regions to exclude from further annotations. These are exons that signify intron-retention (overlapping with two adjacent spliced exons) and thus are excluded as valid exon IDs. These regions are also flagged as intron-retention regions for later probe set annotations.
2. Assembles exons from all transcripts for a gene into discrete exon clusters. If an exon cluster contains multiple exons with distinct boundaries, the exon cluster is divided into regions that represent putative alternative splice sites (Text S1 Figure 1). These splice sites are explicitly annotated downstream. Each exon cluster is ordered and number from the first to the last exon cluster (e.g., E1, E2, E3, E4, E5), composed of one or more regions. These exon cluster/region coordinates and annotations are stored in memory for downstream probe set alignment in the module ExonArrayAffyRules.py.
3. Identifies alternative splicing events (cassette-exon inclusion, alternative 3’ or 5’ splice sites, alternative N-terminal and C-terminal exons, and combinations there in) for all Ensembl and UCSC transcripts by comparing exon cluster and region numbers for all pairs of exons in each transcript (see proceeding sections for more information). Alternative exons/exon-regions and corresponding exon-junctions are stored in memory for later probe set annotation and exported to summary files for creation of databases for the Cytoscape exon structure viewer, SubgeneViewer (currently in development).

Upon completion, ExonArrayAffyRules.py:

1. Imports Affymetrix Exon 1.0 ST probe sets genomic locations, transcript cluster, and mRNA counts from the Affymetrix probe set.csv annotation file (e.g., HuEx-1_0-st-v2.na23.hg18.probeset.csv). Although transcript clusters will be disregarded at the end of the analysis, these are used initially to group probe sets.
2. Transcript cluster genomic locations are matched to Ensembl genes genomic locations (gene start and stop) to identify single transcript clusters that align to only one Ensembl gene for the respective genomic strand. For transcript clusters aligning to more than one Ensembl gene, coordinates for each individual probe set are matched to aligning Ensembl genes, to identify unique matches. If multiple transcript clusters align to a single Ensembl gene, only probe sets with an Affymetrix annotated annotation corresponding to that Ensembl gene, from the probeset.csv file, are stored as proper relationships. This ensures that if other genes, not annotated by Ensembl exist in the same genomic interval, that they will not be inaccurately combined with a nearby Ensembl gene. If multiple associations or other inconsistencies are found, probe set coordinates are matched directly to the exon cluster locations derived in EnsemblImport.py.
3. Once unique probe set-to-Ensembl gene associations have been defined, constitutive probe sets are identified using the Affymetrix mRNA counts provided in the program ExonArrayEnsemblRules.py. The mRNA counts are distributed based on the types of mRNAs they align to (full-length, Ensembl, and EST), where the probe sets with the largest number of high quality mRNA associations are chosen as constitutive. Probe sets for a given gene are ranked based on the: A) number of Ensembl transcripts, B) full-length and C) ESTs associated, in descending order, where multiple associations are required for each annotation type. If all probe sets have the same number of Ensembl and full-length transcript associations, then the number of EST aligning are compared. If no difference in these mRNA assignments exists, no constitutive probe sets are annotated.
4. Each probe sets is then aligned to exon clusters, regions, retained introns, and splicing annotations for that gene. In addition to splicing annotations from EnsemblImport.py, splicing annotations from the UCSC genome annotation file “knownAlt.txt” (found in the same server directory at UCSC as “all_mRNA.txt”) using the program alignToKnownAlt.py, are aligned to these probe sets. If a probe set does not align to an Ensemblmport.py defined exon or intron and is upstream of the first exon or downstream of the last exon, the probe set is assigned a UTR annotation (e.g., U1.1). All aligning probe sets are annotated based on the exon cluster number and the relative position of that probe set in the exon cluster, based on relative 5’ genomic start (e.g., E2.1). This can mean that probe set E2.1 actually aligns to the second exon cluster in that gene in any of the exon regions (not necessarily the first exon region), if it is the most 5’ aligning.
5. These probe set annotations are exported to the directory “AltDatabase/*species*/exon” with the filename “*species* _Ensembl_probe sets.txt”. Exon block and region annotations for each probe set are designated in the AltAnalyze result file.

**Extracting UniProt Protein Domain Annotations Overview**

The UniProt protein database is a highly curated protein database that provides annotations for whole proteins as well as protein segments (protein features or domains). These protein feature annotations correspond to specific amino acid (AA) sequences that are annotated using a common vocabulary, including a class (feature key) and detailed description field. An example is the TCF7L1 protein (<http://www.uniprot.org/uniprot/Q9HCS4>), which has five annotated feature regions, ranging in size from 7 to 210 AA. One of these regions has the feature key annotation “DNA binding” and the description “HMG box”. To utilize these annotations in AltAnalyze, these functional tags are extracted along with full protein sequence, and external annotations for each protein (e.g., Ensembl gene) from the “uniprot_sprot_*taxonomy*.dat” file using the ExtractUniProtFunctAnnot.py program. FTP file locations for the UniProt database file can be found in the file “Config/Default-file.csv” for each supported species. To improve Ensembl-UniProt annotations, these relationships are also downloaded from BioMart and stored in the folder “AltDatabase/uniprot/*species*” as “*species*_Ensembl-UniProt.txt”, which are gathered at ExtractUniProtFunctAnnot.py runtime to include in the UniProt sequence annotation file. These files are saved to “AltDatabase/uniprot/*species*” as “uniprot_feature_file.txt” and “uniprot_sequence.txt”.

**Extracting Ensembl Protein Domain Annotations Overview**
In addition to protein domains/features extracted from UniProt, protein features associated with specific Ensembl transcripts are extracted from the Ensembl database. One advantage of these annotations over UniProt, is that alternative exon changes that alter the sequence of a feature but not its inclusion will be reported as a gain and loss of the same feature, as opposed to just one with UniProt. This is because protein feature annotations in UniProt only typically exist for one isoform of a gene and thus, alteration of this feature in any way will result in this feature being called regulated. Although an Ensembl annotated feature with a reported gain and loss can be considered not changed at all, functional differences can exist do to a minor feature sequence change that would not be predicted if the gain and loss of the feature were not reported.

Three separate annotation files are built to provide feature sequences and descriptions, “Ensembl_Protein”, “Protein”, and “ProteinFeatures” files (“AltDatabase/ensembl/*species*”). The “ProteinFeatures” contains relative AA positions for protein features for all Ensembl protein IDs, genomic start and end locations along with InterPro annotations and IDs. Only those InterPro domains/features with an alignment e-score < 1 are stored for alignment to regulated exons. The “Protein” file contains AA sequences for each Ensembl protein. The “Ensembl_Protein " provides Ensembl gene, transcript and protein ID associations. Data for these files are downloaded and extracted using the previously mentioned EnsemblSQL.py module. The feature annotation source in these files is InterPro, which provides a description similar to UniProt. As an example, see: <http://ensembl.genomics.org.cn/Homo_sapiens/protview?db=core;peptide=ENSP00000282111>, which has similar feature descriptions to UniProt for the same gene, TCF7L1.

## Extracting microRNA Binding Annotations Overview

To examine the potential gain or loss of microRNA bindings sites as the direct result of exon-inclusion or exclusion, AltAnalyze requires putative microRNA sequences from multiple prediction algorithms. These binding site annotations are extracted from the following flat files:

- TargetScan conserved predicted targets (<http://www.targetscan.org/cgi-bin/targetscan/data_download.cgi?db=vert_42>). Gene symbol and putative microRNA associations are extracted (no sequence). The primary gene ID, gene-symbol, is linked to Ensembl based on BioMart downloaded gene-symbol to Ensembl gene annotations (from several Ensembl builds – outdated symbols are often used).
- Miranda human centric with multi-species alignment information was obtained from target predictions organized by Ensembl gene ID (<http://cbio.mskcc.org/research/sander/data/miRNA2003/mammalian/index.html>). A larger set of associations was also pulled from species-specific files (<http://www.microrna.org/microrna/getDownloads.do>), where gene symbol was related to Ensembl gene. Both files provided target microRNA sequence.
- Sanger center (miRBase) sequence was provided as a custom (requested) dump of their version 5 target predictions (<http://microrna.sanger.ac.uk/targets/v5/>), containing Ensembl gene IDs, microRNA names, and putative target sequences, specific for either mouse or human.
- PicTar conserved predicted targets (from Dog to Human) were provided as supplementary data (Supplementary Table 3) at <http://www.nature.com/ng/journal/v37/n5/suppinfo/ng1536_S1.html>, with conservation in human, chimp, mouse, rat, and dog for a set of 168 microRNAs. For mouse, human gene symbols were searched for in the BioMart derived “Mm_ Ensembl_annotation.txt” table after converting these IDs to a mouse compatible format (e.g., TCF7L1 to Tcf7l1). The same was used for aligning to PicTar. The same strategy is used for rat.

Ensembl gene to microRNA name and sequence are stored for all prediction algorithm flat files and directly compared to find genes with one or more lines of microRNA binding site evidence using the program MatchMiRTargetPredictions.py. The flat file produced from this program (“miRBS-combined_gene-target-sequences.txt”) was used by the program ExonSeqSearch.py to search for these putative microRNA binding site sequences among all probe sets from the “*species*_Ensembl_probe set.txt” file built by ExonArrayEnsemblRules.py and probe set sequence from the Affymetrix 1.0 ST probe set fasta sequence file (Affymetrix). Two resulting files, one with any binding site predictions and another required to have evidence from at least two algorithms, are saved to “AltDatabase/*species*/*array_type*/” as “*species*_probe set_microRNAs_any.txt” and “*species*_probe set_microRNAs_multiple.txt”, respectively.

## Inferring Protein-Probe Set Associations Overview

To obtain associations between specific probe sets and proteins, the programs IdentifyAltIsoforms.py was written. The program IdentifyAltIsoforms.py grabs all gene mRNA transcripts and associated exon genomic coordinates from Ensembl and the UCSC Genome Database and compares these to probe set coordinates to find pairs of transcripts (one containing the probe set and another that does not) that have the least number of differing exons (see the section “Protein/RNA Inference Analysis” listed later in this document). These transcript pairs are thus most likely to be similar with exception to the region containing the probe set or critical exon. Once these best matches are identified, corresponding protein sequences for each mRNA are downloaded from Ensembl using EnsemblSQL.py or are downloaded using NCBI webservices via BioPython’s Entrez function.

If protein sequences are unavailable for an mRNA accession, the mRNA sequence for that identifier is downloaded (using the above mentioned services) and translated using the custom IdentifyAltIsoforms.py function “BuildInSilicoTranslations”, which uses functions from the BioPython module to translate an mRNA based on all possible start and stop sites to identify the longest putative translation that also shares either the first or last 5 AA of its sequence with the N-terminus or C-terminus (respectfully) of a UniProt protein.

At this point, only two mRNAs are matched to each probe set, an aligning and non-aligning mRNA. Next, differences in protein feature composition between these two proteins, alternative N or C-terminal sequences, coding sequence and protein length are assessed using ExonAnalyze_module.py and exported to text files (associations and protein sequences) for import when AltAnalyze is run. These two files have the suffix “exoncomp.txt” and are the input for the competitive isoform analysis described in this report. Two analogous files with the suffix “seqcomp.txt”, are derived by identifying the best aligning and non-aligning proteins based on comparison of protein sequence as compared exon composition (e.g. least differences in protein domain composition). This method is referred to as the exhaustive isoform analysis. Additional information on competitive isoform and exhaustive analysis is provided later in this document.

## Annotating Alternative Splicing Events

To predict whether or not a single probe set associates with an alternative splicing or alternative promoter sequence, AltAnalyze uses two strategies; 1) Identify alternative exons/introns based on *de novo* isoform comparison and 2) Incorporating splicing predictions from UCSC’s “known_alt.txt” file.

***De Novo* Splicing Prediction and Exon Annotation**

In order to identify exons with alternative splicing or alternative promoters, AltAnalyze compares all available gene transcripts from the UCSC Genome Database and Ensembl to look for shared and different exons. To achieve this, all mRNA transcripts from UCSC’s species-specific “mRNA.txt” file that have genomic coordinates aligning to a single Ensembl gene and all Ensembl transcripts from each Ensembl build are extracted. Only UCSC transcripts that have a distinct exon composition from Ensembl transcripts are used in this analysis, excluding those that have a distinct genomic start or stop position for the first and last exon respectively (differing 5’ and 3’ UTR agreement), but identical exon-structure.

When assessing alternative splicing, cases of intron-retention are identified first. These regions consist of a single exon that spans two adjacent exons at least one another transcript for that same gene. These retained introns are stored for later analysis, but eliminated as annotated exons. Remaining exons are clustered based on whether their genomic positions overlap (e.g., alternate 5’ or 3’ start sites). Each exon cluster is considered an exon block with one or more regions, where each block and region is assigned a numerical ID based on genomic rank (e.g., E1.1, E1.2, E2.1, E3.1). For each exon in a transcript, the exon is annotated as corresponding to an exon block and region number (Text S1 Figure 1). All possible pair-wise transcript comparisons for each gene are then performed to identify exon pairs that show evidence of alternative exon-cassettes, alternative 3’ or 5’ splice sites or alternative-N or -C terminal exons (Text S1 Figure 1). All transcript exon pairs are considered except for those adjacent to a retained intron. This analysis is performed by comparing the exon block ID and region IDs of an exon and it’s neighboring exons to the exon blocks and regions in the compared transcript. Ultimately, a custom heuristic assigns the appropriate annotation based on these transcript comparisons.

| 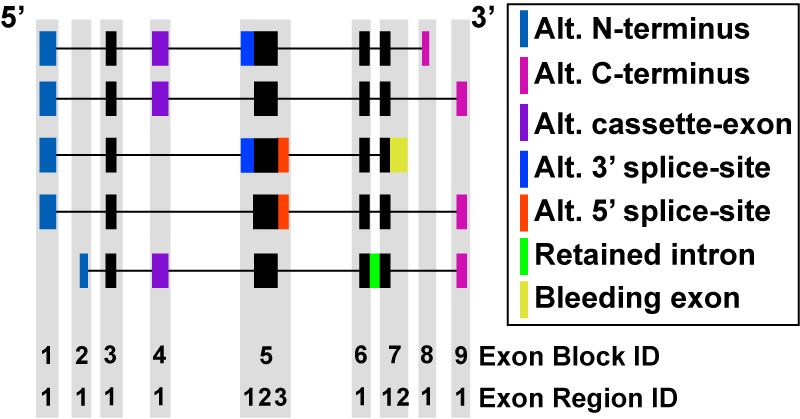 |
| --- |

| **Text S1 Figure 1. Comparison of mRNA Exon Composition**. To determine alternative splicing and alternative promoter regulation, all analyzed transcripts (Ensembl and UCSC) were compared based on exon genomic positions and subsequently annotated by a custom heuristic. Exon block and regions definitions are shown. The different types of alternative exon events are illustrated by different colored exons among five theoretical transcripts for the same gene. |
| --- |

**Incorporating UCSC Splicing Predictions**

In addition to all *de novo* splicing annotations, additional splicing annotations are imported from the UCSC genome database and linked to existing exon blocks and regions based on genomic coordinate overlap. This comparison is performed by the alignToKnownAlt.py module of AltAnalyze (called from EnsemlbImport.py). *De novo* and UCSC splicing annotations are stored along with probe set Ensembl gene alignment data in the file <species>_Ensembl_probesets.txt. These annotations are used by AltAnalyze and DomainGraph.

**Filtering for Alternative Splicing**

AltAnalyze includes the option restrict alternative exon results to only those probe sets predicted to indicate alternative splicing. AltAnalyze considers alternative splicing as any alternative exon annotation other than an alternative N-terminal exon or alternative promoter annotation derived from *de novo* or UCSC genome database annotations.

## Protein/RNA Inference Analysis

**Identifying Alternative Proteins Protein Domains**

In order to identify a single pair of mRNAs that can be used to compare protein features for a given probe set, AltAnalyze compares exon array probe set genomic start and stop positions to all exon genomic start and stop positions, for all mRNA transcripts. Transcripts with exons that contain the probe set genomic coordinates are considered transcript aligning, while all others are considered non-aligning.

If at least one aligning and non-aligning isoform (competitive) are identified for a probe set, all possible aligning and non-aligning pair-wise combinations are compared to find the competitive isoforms with the smallest difference in exon composition. This is accomplished by determining the number of different and common exons each transcript pair contains (based on genomic start and stop of the exon). When comparing the different transcript pairs, the most optimal pair is selected by first considering the combined number of distinct exons in both compared transcripts and second the number of common exons in both compared transcripts. Thus, if one transcript pair has 4 exons in common and 2 exons not in common, while a second pair has 5 exons in common and 3 exon not in common, the prior will be selected as the optimal since it contains less overall differences in exon composition (even though it has less common exons than the other pair). A theoretical example is illustrated in Text S1 Figure 2.


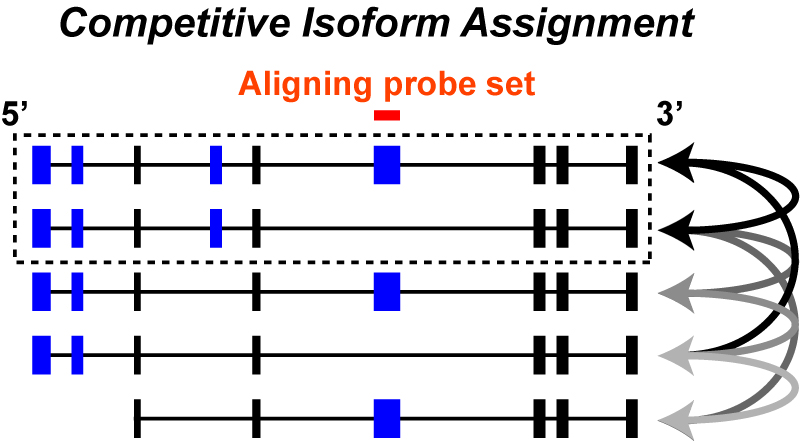


| **Text S1 Figure 2. Comparison of probe set matching and non-matching mRNAs**. A theoretical gene is shown with 9 distinct exons and 5 distinct mRNA transcripts with different exon combinations. Four exons are alternatively expressed in different transcripts (blue exons) while five are common or constitutive to all transcripts (black exons). All possible pair-wise transcript combinations are shown (arrows) between mRNAs that contain and probe set aligning exon and those that do not. Ultimately, a single pair is selected that has the most common exons and the least uncommon exons (dashed box). |
| --- |

Once a single optimal isoform pair has been identified, protein sequence is obtained for each by identifying protein IDs that correspond to the mRNA (Ensembl or NCBI) and if not available, a predicted protein sequence is derived based on *in silico* translation. Although such a protein sequence may not be valid, given that translation of the protein may not occur, these sequences provide AltAnalyze the basis for identifying a possible change in protein size, sequence and domain composition. Domain/protein features are obtained directly from UniProt’s sequence annotation features or from Ensembl’s InterPro sequence annotations (alignment e-value <1) (see previous sections). Any InterPro sequences with a description field or any UniProt sequence annotation feature that is not of the type “CHAIN”, “CONFLICT”, “VARIANT”, “VARSPLIC” and “VAR_SEQ” are examined by AltAnalyze. To compare domain or motif sequence composition differences, the protein sequence that corresponds to the amino-acid start and stop positions of a domain for each transcript is searched for in each of the compared protein isoforms. If the length of a motif sequence is less than 6 amino-acids, flanking sequence is included. If a domain is present in one but not another isoform, that domain is stored as differentially present. To identify differences in protein sequence (e.g., alternative-N-terminus, C-terminus, truncation coding sequence and protein length), the two protein sequences are directly compared for shared sequence in the first and last five residues and comparison of the entire sequences. If the N-terminal sequence is common to both isoforms but there is a reduction in more than 50% of the sequence length, the comparison is annotated as truncated. All of these annotations are stored for each probe set for import into AltAnalyze, with each new database build.

**Direct Domain/Motif Genomic Alignment**

The above strategy allows AltAnalyze to identify predicted protein domains and motifs that are found in one isoform but not the other (aligning to a probe set and not aligning). In addition to these “inferred” domain predictions, that include protein domains/motifs that do not necessarily overlap with the regulated probe set, AltAnalyze includes a distinct set of annotations that only corresponds to domain/motif protein sequence that directly overlaps with a probe set. Alignment of probe sets to InterPro IDs is achieved by comparing probe set genomic coordinates to InterPro genomic coordinates. To obtain InterPro genomic start and end positions are determined by first identifying the relative amino-acid positions of an InterPro region in an Ensmebl protein, finding which exon and at what position the InterPro region begins and ends and finally stroing the genomic position of these relative exon coordinates.

These probe set-InterPro overlaps can be of two types; 1) probe sets whose sequence is present in the domain coding RNA sequence and 2) probe sets whose sequence is not present in the domain coding RNA sequence. The second type of overlap typically occurs in the gene introns. While these associations are typically meaningful, false “indirect” associations are possible. To reduce the occurrences of these false positives, any probe set that aligns to the UTR of an Ensembl gene or that occurs in the first or last exon of an mRNA transcript are excluded. These heuristics were chosen after looking at specific examples that the authors considered to be potential false positives.

**Exhaustive Protein Domain/Motif Analysis**

Very similar to the competitive protein domain/motif analysis is the exhaustive isoform analysis. This feature is currently not available by default in AltAnalyze and requires replacement database files from AltAnalyze support. The purpose of these files is to obtain the most conservative possible domain-level prediction results from the competitive analysis. This done by storing all pair-wise aligning and non-aligning competitive isoform comparisons (Text S1 Figure 1) and then obtaining protein sequence for each transcript, as described in earlier sections and storing all domain/motifs differentially found between all possible competitive protein isoforms. From these stored results, all possible competitive isoforms are themselves compared to find isoforms that ideally show only differences in central regions of the protein (no N-terminal or C-terminal differences), next for those that contain as few possible domain-level predictions and finally for those with the smallest overall differences in protein length. For detailed algorithm information see the IdentifyAltIsoforms module and the function “compareProteinFeaturesForPairwiseComps”.

**Probe set Filtering**

Prior to alternative exon analysis, AltAnalyze was used to remove probe sets that were not deemed as sufficiently expressed. For the two conditions that AltAnalyze compares (e.g., hESC versus CPs), a probe set was removed if neither condition had a mean DABG p value < 0.05. Likewise, if neither condition had a mean probe set intensity > 70, the probe set was excluded from the analysis. For constitutive probe sets (indicating transcription), probe sets were required to have a DABG p value <-0.05 and intensity > 70 in both conditions to be retained. These parameters can be modified in AltAnalyze by the user.

**Splicing Algorithms**

**Splicing Index Method**

This algorithm is described in detail in prior reports [1,2]. In brief, for each probe sets examined, its expression (log_2_) is subtracted from mean expression of all constitutive aligning probe sets to create a constitutive corrected log expression ratio (normalized intensities). Normalized intensities are calculated for each microarray sample, using only data from that sample. To derive the splicing-index value, the mean normalized intensity of all arrays in each control group is subtracted from the experimental. This value is the change in exon-inclusion. A *t* test p-value is calculated (two tailed, assuming unequal variance), by comparing the normalized intensities for all samples between the control and experimental groups. A splicing-index score of -1 indicates a two-fold change in the normalized expression of a probe set in the experiment versus control group, whereas a positive value indicates the opposite.

**MiDAS**

The MiDAS statistic is described in detail in the white paper:

[www.affymetrix.com/support/technical/whitepapers/exon_alt_transcript_analysis_whitepaper.pdf](http://www.affymetrix.com/support/technical/whitepapers/exon_alt_transcript_analysis_whitepaper.pdf). This analysis method is available from the computer program Affymetrix Power Tools or APT. APT uses a series of text files to examine the expression values of each probe set compared to the expression of user supplied gene expression values. Since AltAnalyze uses Ensembl gene IDs rather than Affymetrix transcript clusters, gene expression values are stored with unique numerical gene identifiers for MiDAS analysis. When written, a conversion file is also written that allows AltAnalyze to translate from this arbitrary numerical ID back to an Ensembl gene ID. These relationships are stored in the following files along with the probe set expression values:

| meta-Hs_Exon_cancer_vs_normal.txt | Relates probe set to gene |
| --- | --- |
| gene-Hs_Exon_cancer_vs_normal.txt | Gene expression values (non-log) |
| exon-Hs_Exon_cancer_vs_normal.txt | Probe set expression values (non-log) |
| commands-Hs_Exon_cancer_vs_normal.txt | Contains user commands for APT |
| Celfiles-Hs_Exon_cancer_vs_normal.txt | Relates sample to group |
| probe set-conversion -Hs_Exon_cancer_vs_normal.txt | Relates arbitrary gene IDs back to Ensembl |

When the user selects the option “Calculate MiDAS p-values”, AltAnalyze first exports expression data for selected probe sets to these files for all pair-wise comparisons. Once exported, AltAnalyze interfaces with the APT binary files packaged with AltAnalyze to run MiDAS. MiDAS will create a folder with the pair-wise comparison dataset name and a file with MiDAS p-values that will be automatically read by AltAnalyze and used for statistical filtering (stored in the AltAnalyze program directory under “AltResults/MiDAS”). These statistics are clearly labeled in the results file for each probe set and used for filtering based on the user-defined p-value thresholds (p<0.05 in this analysis).

Note: Different versions of the APT MiDAS binary have been distributed. AltAnalyze is distributed with two versions that report slightly different p-values. The older version (1.4.0) tends to report larger p-values than the most recent distributed (1.10.1). Previous versions of AltAnalyze used version 1.4.0, while AltAnalyze version 1.1 uses MiDAS 1.10.1 that produces p-values that are typically equivalent to those as the AltAnalyze calculated splicing-index p-values (larger).

**Domain/miR-BS Over-Representation Analysis**

A z-score and permutation p-value are calculated to assess over-representation of domains, motifs and microRNA binding sites (miR-BS) found to be effected by alternative exon expression. The z-score is calculated by subtracting the expected number of genes in with a domain/motif or miR-BS meeting the criterion (alternatively regulated with the user supplied thresholds) from the observed number of genes and dividing by the standard deviation of the observed number of genes. This z-score is a normal approximation to the hypergeometric distribution. This equation is expressed as:


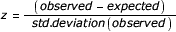

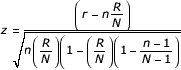


n = All genes associated with a given element

r = Alternatively regulated genes associated with a given element

N = All genes examined

R = All alternatively regulated genes

Once z-scores have been calculated for all domains/motifs and miR-BSs linked to alternatively regulated probe sets, a permutation analysis is performed to determine the likelihood of observing these z-scores by chance. This is done by randomly selecting the same number of regulated probe sets from all probe sets examined and recalculating z-scores for all terms 2000 times. The likelihood of a z-score occurring by chance is calculated as the number of times a permutation z-score is greater than or equal to the original z-score divided by 2000. A Benjamini-Hochberg (BH) correction is used to transform this p-value to adjusted for multiple hypothesis testing.

**Gene Ontology and Pathway Over-Representation Analysis**

To perform advanced pathway and Gene Ontology (GO) over-representation AltAnalyze includes core python modules from the program GO-Elite (version 1.2) in AltAnalyze (<http://www.genmapp.org/go_eite>). GO-Elite performs Gene Ontology (GO) and WikiPathway over-representation using the same algorithms listed for domain/miR-BS over-representation analysis (ORA) (e.g., z-score, permutation p-value, BH p-value calculation). After ORA, all GO terms and pathways are filtered using user-defined statistical cut-offs (z-score, permutation p-value and number of genes changed), with GO terms further pruned to identify a minimally redundant set of reported GO terms, based on hierarchical relationships and ORA scores (see [GO-Elite documentation](http://www.genmapp.org/go_elite/)). All result files normally produced by GO-Elite will be produced through AltAnalyze.

AltAnalyze generates two types of gene lists for automated analysis in GO-Elite; differentially expressed genes and alternatively regulated genes. Criterion for differentially expressed genes are defined by the user in the GO-Elite parameters window (e.g., fold difference > 2 and t-test p < 0.05). In this analysis, the default options were used. All genes associated with alternative exons are used for GO-Elite analysis. Genes with alternative exons that also have alternative splicing annotations can be further selected using the “Filter results for predicted AS” option. Results are exported to the “GO-Elite” directory in the user-defined output directory, while input gene lists can be found in the folders “GO-Elite/input” and “GO-Elite/denominator”. The gene lists for differentially expressed genes have the prefix “GE.” while the alternative exon files have the prefix “AS.”. The appropriate denominator gene files for each is selected by GO-Elite.

When analyzing a new species in AltAnalyze or an unsupported array for the first time, AltAnalyze will build the necessary GO-Elite relational databases to perform both GO and WikiPathway ORA. Probe set, gene and GO relationships will be extracted automatically from the Affymetrix annotation CSV file and gene to WikiPathway relationships from the [http://www.wikipathways.org](http://www.wikipathways.org/) (if relationships are present).

**References**

1. Srinivasan K, Shiue L, Hayes JD, Centers R, Fitzwater S, et al. (2005) Detection and measurement of alternative splicing using splicing-sensitive microarrays. Methods 37: 345-359.

2. Gardina PJ, Clark TA, Shimada B, Staples MK, Yang Q, et al. (2006) Alternative splicing and differential gene expression in colon cancer detected by a whole genome exon array. BMC Genomics 7: 325.
