# Supplementary material for: Alternative Splicing in the Differentiation of Human Embryonic Stem Cells into Cardiac Precursors
Source: PLoS Comput Biol. 2009 Nov 6;5(11):e1000553. doi: 10.1371/journal.pcbi.1000553 (PMC2764345; doi:10.1371/journal.pcbi.1000553)
Supplement: Text S2 — Analysis of sensitivity and specificity of AltAnalyze predictions with a publicly available alternative splicing validation dataset (PTB knockdown). (0.07 MB DOC) [file pcbi.1000553.s005.doc]

# Evaluation of AltAnalyze Predictions With Prior Validation Data

To assess AltAnalyze exon array analysis performance relative to other published approaches, we analyzed published experimental confirmation results for a dataset of splicing factor knockdown (mouse polypyrimidine tract binding protein (PTB) short-hairpin RNA (shRNA)). From two independent analyses [1,2], alternative splicing (AS) for 109 probe sets was assessed in the mouse PTB shRNA dataset by RT-PCR (Supplemental Tables 1,2 and 4 from the referenced study) [2]. Among these, 25 were false positives, one was a true negative, one undetermined and 81 were true positives.

**Summary of Published MADS Results**

In the analysis by Xing et al., alternative exons discovered by multiple splicing array platforms and RT-PCR were examined using a new algorithm named microarray analysis of differential splicing or MADS. MADS implements a modification of the splicing index method on gene expression values obtained using multiple scripts from GeneBase and filtering of probes predicted to hybridize to multiple genomic targets. Using this algorithm, the authors were able to verify AS detected by RT-PCR of mouse Affymetrix Exon 1.0 array data as well as predict and validate 27 novel splicing events by RT-PCR. Using the microarray CEL files posted by Xing and colleagues, we ran AltAnalyze using default options (same as used in the corresponding primary report), which includes quantile normalization via RMA-sketch using AltAnalyze’s interface to Affymetrix Power Tools.

**AltAnalyze Results**

Of the 109 probe sets linked to splicing events characterized by RT-PCR, 78 were analyzed by AltAnalyze. Those probe sets not analyzed by AltAnalyze were either not apart of the AltAnalyze ”core” probe sets or were excluded due to high detection p-value or low expression thresholds. A breakdown of the number of RT-PCR true positive, false positive, undetermined and false negative (out of the 81 documented true positives) is shown for various AltAnalyze filters (Text S2 Table 1).

Of the 78 analyzed probe sets, 26 were called by AltAnalyze to be alternatively regulated (using default parameters), out of 194 probe sets called by AltAnalyze as alternatively regulated. All 26 probe sets were annotated as true positives according to the published RT-PCR data. Although 17 probe sets were RT-PCR false positives among the 78 probe sets with RT-PCR data, only one false positive was considered to be alternative regulated by AltAnalyze with any of the AltAnalyze filters alone (splicing-index p<0.05 alone without additional default options). The MADS algorithm was able to validate AS for 27 novel splice events corresponding to 41 probe sets. Of these 41 probe sets, 33 were examined by AltAnalyze and 23 were considered alternatively regulated.

**Conclusions**

This analysis suggests that AltAnalyze analysis using default parameters produces conservative results with high specificity (100% true positives in this analysis) with reasonable sensitivity (~42% that of MADS). For smaller datasets, such as the PTB knock-down comparison, the decreased sensitivity results will have a significant impact on the number of true splicing events detected, however, for larger datasets with thousands of regulated probe sets, AltAnalyze is likely to reduce the number of false positives and reduction in overall noise. It is important to note however, for the MADS analysis only a p-value threshold was used and for AltAnalyze, both a MiDAS and splicing-index p-value in addition to splicing-index fold change thresholds were used. For these analyses, we have not filtered probe sets based on association with annotated splicing events (see Materials and Methods for description), which should further decrease false positives. If probe sets with annotated splicing events are filtered out, 20 versus 26 true positives will remain.

Although a false positive rate of up-to 50% has been reported with the conventional splicing-index implementation (e.g., using the Affymetrix ExACT software) [2], AltAnalyze’s analysis differs in several ways. First, in this analysis RMA-sketch was used as the method for quantile normalization. After obtaining expression values for probe sets (no low level filtering currently implemented), probe sets for each of the two biological groups are filtered based on two main parameters: DABG p-value and mean expression filters. Any probe set with a DABG p-value > 0.05 in both biological groups or mean expression < 70 are excluded.

Additional AltAnalyze specific parameters relate to how probe set to gene associations are obtained, which probe sets are selected for analysis and how probe sets are selected for calculation of gene expression. Unlike ExACT, probe set to gene association are via genomic coordinate alignment to Ensmebl/UCSC mRNA transcripts for unique Ensembl genes rather than to Affymetrix transcript clusters. This process ensures that a probe set only aligns to one Ensembl gene. Probe sets can align to an exon, intron or UTR of a gene. Any probe set aligning to an analyzed mRNA is used for analysis in the AltAnalyze “core” set along with any Affymetrix annotated “core” probe set. To determine gene expression from the exon-level a two-step method is employed. First, probe sets that are most over-represented among mRNAs or mRNAs and ESTs (associations from Affymetrix probe set annotation file) are selected as constitutive. Next if constitutive probe set is “expressed” in both biological groups (using the DABG and mean expression filters listed above), the probe set is retained for constitutive expression calculation. If more than one “expressed” constitutive probe set is present, the mean expression of all constitutive probe sets for each array is calculated. As a final step, probe sets with an associated gene expression difference between the two array groups greater than 3 are not reported. These analysis steps result in a unique splicing-index, splicing-index *t* test p-value and MiDAS p-value calculation from other analysis methods.

Since this validation set provides a limited test case for analysis of type I (false positive) and type II (false negative) errors, the AltAnalyze algorithms will continue to be assessed as additional validation data is made available. Future implementations will likely include “low-level” analyses that reduce the occurrence of type I errors (e.g., elimination of expression data for specific probes that introduce additional noise). However, this data supports the concept that AltAnalyze produces conservative results with a level of confidence.

|  |  |  |  | AltAnalyze RMA Analysis | | | | |
| --- | --- | --- | --- | --- | --- | --- | --- | --- |
|  | **All** | **MADS p<0.05** | **MADS p<0.01** | **All** | **SF>2** | **SP <0.05** | **SP|MP <0.05** | **SF>2, GEF<3, SP|MP <0.05** |
| ***TP*** | 81 | 62 | 55 | 60 | 30 | 36 | 32 | 26 |
| ***FP*** | 25 | 6 | 2 | 17 | 0 | 1 | 0 | 0 |
| ***UD*** | 1 | 1 | 1 | 1 | 0 | 1 | 1 | 0 |
| ***FN*** | 0 | 19 | 26 | 21 | 51 | 45 | 49 | 55 |

| **Text S2 Table 1 - Analysis of PTB shRNA verified splicing events with AltAnalyze.** The number of probe sets matched to different RT-PCR absence or presence calls for AS from an analysis of mouse PTB shRNA knockdown of a neuroblastoma cell line compared to empty vector shRNA knockdown using the Affymetrix Mouse 1.0 Exon array [12]. Results are shown for all probe sets (All), MADS p<0.05 or p<0.01 (after removing cross-hybridizing probes), AltAnalyze “core” probe sets (All) analyzed by RT-PCR, splicing-index fold change >2, splicing-index t-test p (SP) <0.05, MiDAS p (MP) <0.05 or combination of all three options (SF>2 & SP & MP <0.05 – default option) along with gene expression filtering (GEF) of < 3 fold, for true positive (TP), false positive (FP), un-determined (UD), and false negative (FN) RT-PCR results. False negative probe set counts are relative to all original true positives experimentally identified, independent of whether they were considered by AltAnalyze. |
| --- |

**References**

1. Boutz PL, Stoilov P, Li Q, Lin CH, Chawla G, et al. (2007) A post-transcriptional regulatory switch in polypyrimidine tract-binding proteins reprograms alternative splicing in developing neurons. Genes Dev 21: 1636-1652.

2. Xing Y, Stoilov P, Kapur K, Han A, Jiang H, et al. (2008) MADS: a new and improved method for analysis of differential alternative splicing by exon-tiling microarrays. Rna 14: 1470-1479.
